# Barriers and Facilitators of Implementing Cognitive Behavioral Therapy: A Systematic Review Based on the Consolidated Framework for Implementation

**DOI:** 10.1155/sci5/2693791

**Published:** 2025-11-10

**Authors:** Varshini R. J., Edlin Glane Mathias, R. Sai Bhavana, Sanjay P. Patil, Rajesh Kamath

**Affiliations:** ^1^Department of Healthcare and Hospital Management, Prasanna School of Public Health, Manipal Academy of Higher Education, Manipal, India; ^2^Department of Health Technology and Informatics, Prasanna School of Public Health, MAHE, Manipal, India

**Keywords:** cognitive behavioral therapy, consolidated framework for implementation research (CFIR), implementation barriers, implementation facilitators, mental health interventions, systematic review

## Abstract

Cognitive behavioral therapy (CBT) is currently considered the gold standard of treatment, with its evidence-based framework widely used in healthcare. However, its implementation in real-world settings faces numerous challenges. Understanding the barriers and facilitators of CBT implementation is essential to improving its accessibility and effectiveness. A systematic search was conducted across multiple databases from 20/9/2024 to 10/10/2024, including PubMed, CINAHL, ProQuest, and Web of Science. Studies published in English from 1994 to 2024 were included. This systematic review was reported using the guidelines of Preferred Reporting Items for Systematic Reviews and Meta-Analyses (PRISMA) and registered at the International Prospective Register of Systematic Reviews (Registered No. CRD42024570477). Qualitative, quantitative, and mixed-method studies were analyzed to identify key implementation challenges and enablers. Data extraction and quality assessments were performed using the Mixed Methods Appraisal Tool (MMAT) and the JBI critical appraisal tools. A total of 32 studies with 2082 participants were included in this review. Common barriers to CBT implementation included limited training opportunities for clinicians, high caseloads, organizational constraints, resistance to change, and inadequate funding. Digital literacy challenges and technical issues further hindered the adoption of internet-based CBT (iCBT). Facilitators included strong leadership support, structured training programs, stakeholder engagement, and the integration of digital and step-care approaches to enhance accessibility. The findings highlight the importance of addressing systemic, organizational, and individual-level barriers to improve the implementation of CBT. Strengthening clinician training, increasing institutional support, and leveraging digital solutions can enhance the accessibility and sustainability of CBT interventions. This review provides practical insights for policymakers, mental health professionals, and researchers working toward optimizing CBT implementation in diverse settings.

## 1. Introduction

People with mental disorders experience significantly reduced life expectancy, with an average of 14.7 years lost compared to the general population [1]. In 2019, disability-adjusted life years (DALYs) attributable to mental disorders accounted for 5.30% of global DALYs, up from 3.46% in 1990 [2]. The global impact of mental disorders, measured as the number of years lost due to illness or early death (DALYs), is higher among women than men. The age-standardized DALY rate for mental illnesses was 1426·5 per 100,000 population for males and 1703·3 per 100,000 population for females [3]. One out of every eight individuals, or 970 million people worldwide, suffered from a mental disorder in 2019, with anxiety and depression being the most common. The COVID-19 pandemic also led to a significant increase in the number of people suffering from anxiety and depression in 2020. Preliminary forecasts suggest that these numbers have continued to rise; in just one year, anxiety and major depressive disorders have increased by 26% and 28%, respectively [4]. Treatment and management of these illnesses vary from pharmacological approaches to therapies such as psychotherapy and behavioral therapy [5].

Cognitive behavioral therapy (CBT) is a type of psychotherapy that focuses on addressing unhealthy thoughts and unhelpful behaviors [6]. CBT is currently considered the gold standard of treatment, with its evidence-based framework widely used in healthcare [7]. Approximately 75% of those who undergo psychotherapy report some benefit, including fewer sick days, less disability, fewer medical problems, and increased work satisfaction [8]. Response rates to CBT ranged from 38% for obsessive–compulsive disorder (OCD) to 82% for body dysmorphic disorder. In contrast, response rates for the waitlist groups ranged from 2% for bulimia nervosa to 14% for generalized anxiety disorder (GAD). Out of 11 studies comparing response rates between CBT and other treatments or control conditions, 7 found that CBT had a higher response rate than the comparison conditions, which included pharmacological approaches, relaxation therapy, supportive therapy, psychodynamic therapy, placebo/control treatments, treatment as usual (TAU), and waitlist treatment [9].

Despite its effectiveness and potential benefits, CBT is often underutilized in practice settings [10]. Barriers include high productivity demands, cognitive deficits, family issues among adolescents, frequent changes in clinician caseloads, and organizational factors such as inadequate staffing and a challenging work environment. Additionally, some clinicians' attitudes toward research and concerns about increased workload also hinder its implementation [11].

Conversely, facilitators of CBT implementation include clinician training and supervision, clinician motivation and competence, strong consumer engagement, and adaptable training manuals that cater to individual client needs. Effective implementation requires addressing barriers while leveraging facilitators at multiple levels, including those related to consumers, clinicians, organizations, and environmental factors [11].

The Consolidated Framework for Implementation Research (CFIR) identifies potential barriers and facilitators in the implementation and evaluation of research studies. The CFIR guide has been specifically utilized by researchers in mental health care to enhance the implementation and evaluation of interventions. It offers a set of constructs across five domains that assist in pinpointing these barriers and facilitators in implementation design. The five domains include: (1) characteristics of available intervention (e.g., need for training manuals and other dissemination tools must be created that allow for flexibility in the treatment), (2) inner setting (e.g., productivity demands, caseload changes, and organizational factors such as inadequate staffing support and the overall work environment reflect issues within the internal setting of the clinic that hinder CBT implementation), (3) outer setting (e.g., strong consumer engagement and advocacy for CBT among adolescents and their families reflect the external demand and support that can facilitate implementation efforts), (4) characteristics of individuals (e.g., an individual's characteristics such as beliefs, self-efficacy, and personal attributes that may affect help-seeking behaviors, clinician motivation, and competence are crucial individual characteristics that facilitate the adoption of CBT), and (5) implementation process (e.g., the flexibility of training manuals and the provision of ongoing supervision are critical components of the implementation process that help integrate CBT into practice. Issues such as productivity demands and caseloads reflect challenges in the planning and implementation process). All five domains interact within this framework for this study to identify the barriers and facilitators of CBT and implementation of the intervention [11, 12].

This systematic review aims to synthesize existing evidence from all related stakeholders regarding barriers and facilitators of implementing CBT. CFIR provides a standardized framework for analyzing and assessing facilitators and barriers related to CBT. This helps develop effective interventions and increase the utilization of mental health resources.

## 2. Research Methodology

This systematic review was reported using the guidelines of Preferred Reporting Items for Systematic Reviews and Meta-Analyses (PRISMA) and registered at the International Prospective Register of Systematic Reviews (Registered No. CRD42024570477). The PROSPERO registration record for this systematic review, including the registered review protocol, is provided in Supporting File 1.

### 2.1. Inclusion/Exclusion Criteria

The Patient/Population, Intervention, Comparison, and Outcome (PICO) framework was followed to address the review question.

### 2.2. Criteria for Including Studies in the Review Population

This review includes all related stakeholders (e.g., patients, mental healthcare providers, and policymakers).

### 2.3. Interventions

This review includes interventions that focuses on patient experiences and perspectives: This includes factors influencing their understanding, acceptance, and engagement with interventions. Provider experiences and perspectives: This involves facilitators and barriers to implementing interventions, perceived effectiveness, and fit within workflow.

Implementation challenges and facilitators: Studies that explore aspects like accessibility, training needs, stakeholder involvement, and ongoing support during implementation.

### 2.4. Outcomes

Barriers and facilitators of implementing CBT based on the CFIR.

### 2.5. Types of Studies

This review includes qualitative, quantitative studies and mixed methodology studies. This review included qualitative, quantitative, and mixed-methods studies that reported on the barriers and/or facilitators to implementing CBT. However, given the nature of the synthesis guided by the CFIR and employing a deductive thematic synthesis approach, only studies providing descriptive implementation data amenable to qualitative coding were analyzed in depth. This included qualitative and mixed-method studies and select quantitative studies that reported implementation-related outcomes in narrative or categorical form (e.g., surveys reporting frequency of barriers or facilitators). Studies that solely provided numerical effectiveness outcomes without descriptive implementation data were excluded.

### 2.6. Search Strategy

The search strategy aimed to find published studies. A three-step search strategy was utilized in this review. An initial limited search of PubMed (NCBI), CINAHL (EBSCO), PROQUEST, and Web of Science was undertaken, followed by analysis of the text words contained in the title and abstract, and of the index terms used to describe articles. Studies published in English from 1994–2024 were considered for inclusion in this review. The detailed search strategy, including databases searched, time periods, and full search strings, is provided in Supporting File 2.

## 3. Initial Keywords

Search terms—These terms aimed to represent the primary concepts of “implementing,” “Cognitive behavior therapy,” and “barriers” or “facilitators.” Keywords were generated for each of these concepts by examining the terminology used in review papers in the implementation literature and a thesaurus to locate synonyms. In addition, the keywords were combined with standard MeSH terms from the PubMed and Cochrane databases.

### 3.1. Selection of Studies

The selection process was guided by the PICO framework and involved a systematic screening of titles, abstracts, and full-text articles. Studies were included if they involved relevant stakeholders such as patients, mental healthcare providers, and policymakers, and focused on the implementation of CBT. Eligible studies explored patient and provider experiences, implementation challenges and facilitators, and reported outcomes aligned with the CFIR. Both qualitative and mixed-methods studies published in English were considered. Exclusion criteria included studies that focused solely on the clinical effectiveness of CBT without addressing implementation processes, those unrelated to mental health or cognitive behavioral approaches, and non–peer-reviewed literature, such as editorials, opinion pieces, or conference abstracts. Screening of selected articles—Screening and selection of titles, abstracts, and full text were conducted independently by two authors with the help of RAYYAN database [13]. Discrepancy between authors was resolved by discussion.

The selection process involved multiple levels of screening based on predefined eligibility criteria. Full-text articles were excluded for the following specific reasons:

• Wrong study design: Studies that did not use a qualitative, quantitative, or mixed-method approach to investigate implementation processes.
  For example, editorials, commentaries, opinion pieces, and case reports were excluded.
• Wrong population: Studies that did not involve relevant stakeholders such as patients, mental health providers, caregivers, or policymakers involved in CBT delivery.
  For instance, studies conducted solely among pharmacologists, undergraduate students, or populations outside the mental health domain were excluded.
• Wrong outcome: Studies that focused only on the clinical effectiveness or efficacy of CBT (e.g., symptom reduction, comparative treatment outcomes), without reporting any barriers or facilitators related to CBT implementation.

For example, an RCT comparing CBT vs. medication for anxiety that reported symptom scores but provided no information on delivery challenges or stakeholder perspectives was excluded.

### 3.2. Data Extraction

A predesigned Excel sheet was prepared by the authors. Five studies were pilot tested prior to data extraction of the included studies. Data were extracted from papers included in the review using MS Excel. The data extracted include specific details about the intervention, populations, study methods, and outcomes that are significant to the review question and specific objective.

### 3.3. Assessment of Methodological Quality

Three independent reviewers assessed methodological quality utilizing the standardized Joanna Briggs Institute (JBI) critical appraisal tool and the Mixed Methods Appraisal Tool (MMAT) appraisal instrument. Mixed-method publications chosen for retrieval were evaluated for methodological validity before inclusion in the review using the MMAT. Before being included in the review, qualitative papers selected for retrieval were appraised for methodological validity using the JBI appraisal tool [14, 15].

### 3.4. Data Synthesis

Data synthesis involved the aggregation or synthesis of findings to generate a set of statements that represent that aggregation through assembling the findings rated according to their quality and categorizing them based on similarity of meaning. The findings are presented in narrative form, and tables and figures were also incorporated to organize and visually represent the study findings. The themes were identified for the data analysis of qualitative studies and presented in the narrative form. We adopted a deductive thematic synthesis approach guided by the CFIR to analyze and organize findings from the included studies. The CFIR framework, which comprises five major domains (Intervention Characteristics, Outer Setting, Inner Setting, Characteristics of Individuals, and Process), along with associated constructs, served as the a priori coding structure for data extraction and synthesis. One reviewer independently coded each of the 32 included studies by mapping the reported implementation barriers and facilitators to relevant CFIR domains and constructs. A coding matrix was developed to systematically capture and classify extracted data across studies. The process involved thoroughly reading the results and discussion sections of each study to identify implementation-related determinants and assigning them to the most appropriate CFIR construct. Ambiguous or overlapping data were resolved by consulting the CFIR codebook and in discussion with the research team. The synthesized findings were then organized and presented according to the five CFIR domains to identify commonly reported barriers and facilitators. Frequencies of reported constructs were tabulated to highlight patterns across studies and guide interpretation. Although studies with diverse methodologies were eligible for inclusion, the synthesis focused on findings with rich descriptive content. Therefore, quantitative studies that lacked qualitative or narrative reporting on implementation processes were not included in the thematic synthesis.

## 4. Results

### 4.1. Selection and Inclusion of Studies

**Figure 1:**
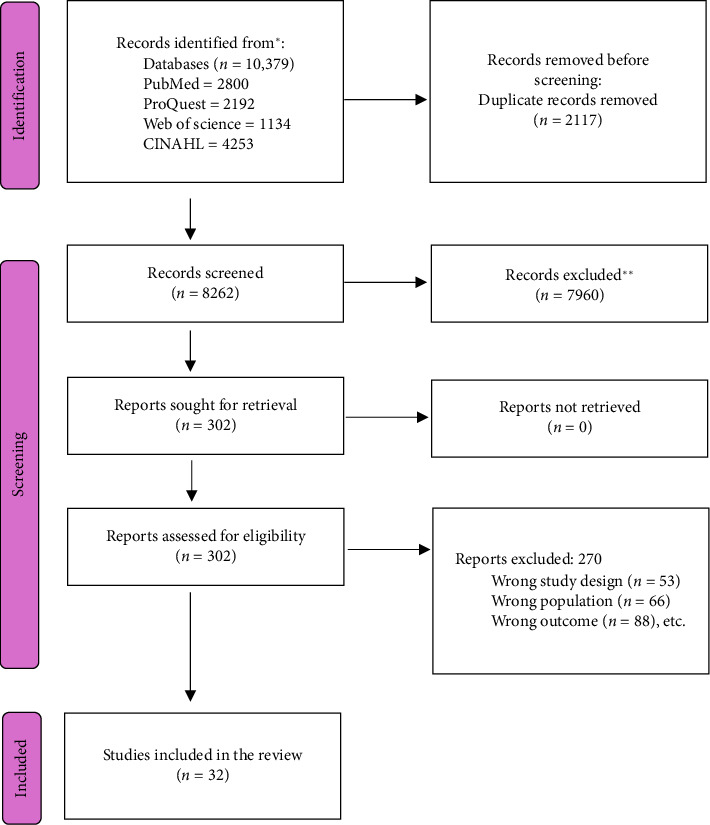
PRISMA flow diagram.

The study selection process adhered to PRISMA [16] guidelines and is summarized as follows. A total of 10,379 records were identified from database searches, including PubMed (*n* = 2800), ProQuest (*n* = 2192), Web of Science (*n* = 1134), and CINAHL (*n* = 4253). After removing 2117 duplicate records, 8262 records were screened for eligibility. Of these, 7960 records were excluded based on title and abstract screening. Subsequently, 302 reports were sought for full-text retrieval, of which 253 could not be retrieved. The remaining 49 reports were assessed for eligibility, resulting in the exclusion of 17 studies due to reasons, such as wrong study design (*n* = 3), wrong population (*n* = 2), and wrong outcomes (*n* = 12). Ultimately, 32 studies included in the final review were selected based on rigorous eligibility criteria. The description of the study selection is provided in Figure 1.

### 4.2. Characteristics of Included Studies

Thirty-two studies included in this systematic review had a combined total sample size of 2082 participants, with diverse populations and settings. The sample sizes ranged from small focus groups of 12 individuals to large cohorts of 320 primary care patients. Participants included 107 psychologists working in medical settings; 148 individuals comprising providers, administrators, and patients in community mental health settings; and 79 patients undergoing CBT for psychosis. Additionally, there were 125 patients diagnosed with chronic fatigue syndrome, 320 primary care patients with chronic cardiopulmonary diseases experiencing depression and anxiety, and 82 adults diagnosed with social anxiety or panic disorders. Unique groups included adjudicated teens in residential treatment facilities (129), 86 community-based clinicians, and 59 participants across nine focus groups. Several studies focused on mental health professionals, such as 124 clinicians in community settings, 145 participants in therapeutic roles, and 243 mental health professionals in diverse care settings. Other notable groups included cancer patients (10), behavioral health providers (18) in integrated primary care settings, stakeholders in the UK's Improving Access to Psychological Therapies program (IAPT), and therapists and managers in outpatient psychiatric care. The populations spanned a range of clinical and community environments, including school-based service providers, primary care clinics, residential treatment programs, and veterans' health systems, offering a comprehensive perspective on psychological and therapeutic interventions across diverse contexts. Detailed insights into characteristics of included studies are given in Table 1.

**Figure 2:**
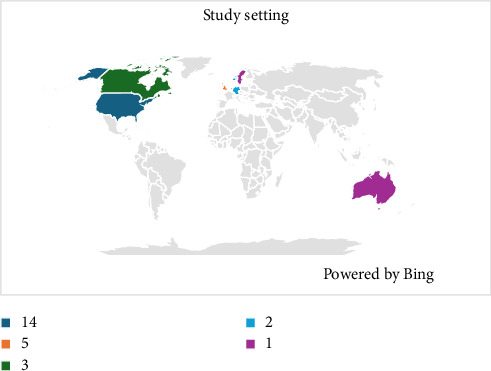
Distribution of study setting.

Studies for this systematic review were conducted across various countries as shown in Figure 2: the Netherlands, the United States, Germany, Norway, the United Kingdom, Canada, Australia, Sweden, and a combined setting of Albania and Kosovo.

#### 4.2.1. Netherlands

One study surveyed psychologists working in medical settings, finding that limited access to internet-based CBT (iCBT), insufficient training, and organizational barriers hindered uptake [17]. Another study conducted a cost and outcomes analysis of CBT for chronic fatigue syndrome, reporting feasibility and patient satisfaction as key facilitators, though implementation was resource-intensive [18]. A third study explored therapist and manager perspectives on guided iCBT for chronic pain and fatigue, emphasizing the importance of flexible delivery formats, management support, and staff readiness for successful integration [19].

#### 4.2.2. United States

One study identified stakeholder-perceived barriers to exposure-based CBT in community mental health, including stigma and resource constraints [20]. Another investigated brief CBT in primary care, highlighting the need for adequate clinician training and addressing organizational stigma [21]. A third study evaluated trauma-focused CBT (TF-CBT) for adjudicated teens, showing promising outcomes but noting difficulties with staff buy-in and resource allocation [22]. One study examined training augmented with self-care, finding that therapist well-being was both a facilitator and a challenge to implementation [23]. Another investigated school-based TF-CBT, identifying systemic barriers such as high caseloads and competing demands [24]. One study evaluated stepped care for psychosis, reporting acceptability but also challenges around feasibility in real-world practice [25]. Another focused on provider perspectives in VA primary care, reporting logistical barriers but also highlighting existing strategies such as adaptation of workflows [26]. One study explored integrated primary care, emphasizing difficulties in adopting CBT-based anxiety interventions due to time pressures and resource gaps [27]. Another examined CBT for youth anxiety, noting that lack of community resources and inconsistent supervision impeded implementation [28]. One study evaluated school-based computer-assisted CBT, illustrating contextual challenges around technology access and teacher involvement [29]. Another studied CBT for criminogenic thinking in the VA system, highlighting the role of organizational support in overcoming stigma and skepticism [30]. One study described implementation of the CALM intervention for anxiety disorders, reporting strong stakeholder engagement but challenges with sustaining fidelity [31]. Another evaluated access to TF-CBT for care-experienced youth, identifying systemic and policy-level barriers [32]. Finally, one study explored clinicians' perspectives on CBT in community settings, emphasizing that ongoing supervision and leadership support were essential for sustaining delivery [33].

#### 4.2.3. Germany

One study examined CBT for psychosis, finding that the therapeutic alliance was a critical facilitator for change processes [34]. Another explored therapists' perspectives on blended therapy, identifying technological barriers and integration challenges but also noting benefits in flexibility and scalability [35].

#### 4.2.4. Norway

One study evaluated CBT for social anxiety and panic disorder, showing that therapist competence and working alliance strongly predicted positive outcomes [36]. Another investigated general practitioners' experiences with guided iCBT for depression, reporting that time constraints, limited training, and skepticism impeded integration into primary care [37].

#### 4.2.5. United Kingdom

One study explored the dissemination of brief CBT for voices, identifying stigma and skepticism as major barriers [38]. Another conducted a peer audit within assertive outreach teams, finding resistance to CBT and insufficient staff training as challenges [39]. One applied the normalization process theory to CBT for psychosis, identifying cultural and systemic barriers but highlighting the framework's utility in guiding implementation [40]. Another examined stakeholder experiences with internet-delivered CBT, pointing to digital literacy gaps and organizational challenges as key issues [41]. Finally, one investigated TF-CBT for young people with care experience, underscoring systemic obstacles in service access and resource allocation [32].

#### 4.2.6. Canada

One study examined stepped-care CBT for insomnia in cancer patients, noting that fatigue, competing health demands, and patient burden hindered implementation [42]. Another reported on a publicly funded iCBT program during COVID-19, with patients and therapists highlighting engagement, privacy, and accessibility as central issues [43]. A third evaluated transdiagnostic CBT in child welfare programs, identifying the need for trauma-informed approaches and stronger interdisciplinary collaboration [44].

#### 4.2.7. Australia

One study assessed commencement and persistence with a web-based CBT intervention for insomnia, finding that digital literacy and technical difficulties were barriers, whereas flexibility and accessibility facilitated engagement [45].

#### 4.2.8. Sweden

One study reported on the implementation of internet-delivered CBT for insomnia in psychiatric health care, applying the NASSS framework and highlighting strong management support and adaptability of the intervention as facilitators [46].

#### 4.2.9. Albania and Kosovo

One study examined organizational readiness for iCBT for depression, reporting that digital infrastructure limitations and cultural stigma were barriers, while cross-border collaboration and institutional commitment facilitated implementation [47].

The studies analyzed include a diverse range of methodologies aimed at exploring various aspects of interventions and programs. The studies in the review provided insights from multiple perspectives, with qualitative studies contributing rich, in-depth narratives on clinician and patient experiences. These qualitative findings highlighted the subjective challenges and support encountered during the adoption of CBT. Cross-sectional studies contributed data on the prevalence of implementation barriers and the factors associated with successful uptake. Quasiexperimental studies and randomized controlled trial (RCT) studies provided evidence on the effectiveness of interventions aimed at overcoming implementation challenges, while mixed-method studies combined both quantitative and qualitative insights, offering a comprehensive view of the implementation landscape.

### 4.3. Quality Assessment

A comprehensive quality assessment was conducted on the 32 studies included in this review, of which 26 were determined to be of high quality and 6 of low quality. Methodological rigor was evaluated using established appraisal tools tailored to each study design: the JBI Critical Appraisal Checklist for Analytical Cross-Sectional Studies, the JBI Critical Appraisal Checklist for Randomized Controlled Trials, the JBI Critical Appraisal Checklist for Qualitative Research, and the MMAT for mixed-method studies [14, 15]. The high-quality studies consisted of 3 RCT studies, 20 qualitative studies, and 3 mixed-method studies. Conversely, the low-quality studies included 2 cross-sectional studies, 1 RCT, 1 quasiexperimental study, and 2 qualitative studies. This rigorous appraisal ensured the reliability and validity of the included studies, thereby enhancing the overall credibility of the research findings.

Among the high-quality studies, several RCTs such as those by Cully [21], Haug [36], and Cohen [22] demonstrated methodological rigor through robust randomization, allocation concealment, and appropriate blinding procedures. In addition, a large number of qualitative studies, including those by Shepardson [28], Savard [40], and Kopelovich [29], showed strong alignment between their research questions, data collection, and analytical methods, thereby meeting criteria for transparency and coherence. Mixed-methods studies such as Harrison [30], Connors [46], and Kopelovich [44] also scored highly, with clear integration of quantitative and qualitative findings that strengthened the reliability of their conclusions.

In contrast, six studies were considered low quality. For example, two cross-sectional surveys (IJzerman and van der Vaart [17] and Wolitzky-Taylor [20]) suffered from insufficient reliability and validity in measurement tools, as well as limited adjustment for confounding factors. A quasiexperimental study by Scheeres [34] was downgraded due to the absence of a control group and incomplete follow-up, which reduced its internal validity. Similarly, one RCT (Cully [18]) failed to implement adequate blinding procedures, and two qualitative studies (Williams [39] and Hazell [43]) lacked sufficient methodological transparency and did not report ethical approval, resulting in their classification as low quality.

Overall, the methodological strengths of the majority of included studies enhance the credibility of the synthesized findings, while limitations in a few weaker studies were carefully considered during interpretation. Two cross-sectional studies were rated as low quality, though they clearly defined inclusion criteria and provided detailed descriptions of the study subjects and settings, and they lacked validity and reliability in measuring exposure and outcomes. Additionally, confounding factors were not adequately identified or addressed in these studies, which further weakened their methodological rigor. The quasiexperimental study reveals several limitations that contributed to its low quality, while it was clear in the study what constituted the “cause” and “effect,” the absence of a control group significantly weakened the design, as there were no comparisons with a group not receiving the intervention. The absence of key elements such as a control group, unreliable outcome measurement, and complete follow-up contributed to its classification as low quality. Three of the four RCT studies assessed were rated as high quality, while one was categorized as low quality based on their adherence to key RCT principles. The high-quality studies excelled in multiple domains, including randomization, allocation concealment, and ensuring that participants, those delivering the treatment, and outcome assessors were blinded to treatment assignment. These studies also adhered to appropriate statistical analysis and followed the standard RCT design, with deviations clearly accounted for in the analysis. On the other hand, the low-quality study used randomization, but it did not blind participants or those delivering the treatment, nor was allocation concealed, and there were issues with treatment groups not being treated identically, and follow-up was incomplete, with no analysis of the differences between the groups regarding follow-up. The lack of blinding and incomplete follow-up contributed to the study's low quality despite other strengths in its design and outcome measurement. The quality assessment of the qualitative studies reveals generally strong adherence to key research principles, with 20 studies rated as high quality (studies with more than or equal to seven “YES” were considered of high quality and below seven are considered low quality). A consistent strength across these studies is their alignment between the research methodology, research questions, data collection methods, and the analysis of data. Two studies demonstrated notable shortcomings in research transparency and lacked ethical approval from an appropriate body, leading to their classification as low quality. Three mixed-method papers were rated high quality (studies with more than or equal to 15 “YES” were considered of high quality) due to strong performance in qualitative studies and mixed-methods studies. They had clear research questions, well-structured data collection, and effective integration of qualitative and quantitative methods. Their rigorous study design ensured methodological coherence and thorough analysis. While quantitative nonrandomized studies showed some variability, the overall methodological strength secured their high-quality rating. The quality assessment results are presented in Supporting file 3.

### 4.4. Risk Bias Assessment

A thorough risk of bias assessment was conducted for all included studies using validated tools tailored to their respective designs. The ROBINS-I tool was utilized to assess the risk of bias in nonrandomized studies [48]. Among these, one quasiexperimental study exhibited a serious risk of bias, while the qualitative studies included 20 with moderate risk, one with low risk, and one with serious risk of bias. The quasiexperimental study faces substantial concerns due to multiple domains with moderate and serious risks of bias, particularly in handling missing data, measurement of outcomes, and deviations from intended interventions [18]. The 22 qualitative studies demonstrated varied risk of bias profiles. Twenty were judged as moderate overall, one as low, and one as serious. The assessments were based on seven domains (confounding, classification of interventions, participant selection, deviations from intended interventions, missing data, outcome measurement, and reporting). Below, we summarize how each study's domain-specific ratings informed its overall categorization and its contribution to the synthesis. Mostly, moderate risk ratings, particularly for participant selection and outcome measurement, led to an overall moderate risk classification. The study nevertheless provides valuable insight into provider-level challenges within the VA system [26]. Low risk across most domains, but moderate in participant selection and reporting bias resulted in a moderate overall risk. The dual perspective from clinicians and service users adds depth to understanding dissemination barriers [38]. Moderate risk of bias from participant selection and missing data, despite otherwise low domain ratings, led to a moderate overall judgment. This supports interpretation of patient engagement with digital CBT [45]. The presence of moderate risk in participant selection, alongside otherwise low domain ratings, produced a moderate risk overall. It adds context regarding cultural and systemic issues in web-based CBT adoption [37]. Moderate risks in participant selection, missing data, and reporting explain its moderate overall classification. This study highlights practical barriers to CBT delivery in integrated primary care [27]. Serious risk ratings across multiple domains (confounding, participant selection, outcome measurement, and reporting) resulted in an overall serious risk. Its methodological weaknesses limited its weight in the synthesis, though it identifies unique challenges in assertive outreach teams [39]. Mostly, low ratings, with moderate bias in participant selection and outcome measurement, led to a moderate overall rating. This study informs patient-level implementation challenges in oncology [42]. Moderate risk of bias from participant selection and outcome measurement resulted in a moderate overall judgment, though the study offers valuable therapist perspectives two years after training [28]. Moderate participant selection and outcome measurement issues contributed to a moderate risk classification, but the application of the NPT framework strengthens interpretation [40]. Low risk across most domains, with moderate risk in participant selection, gave a moderate overall rating. Its contribution lies in highlighting readiness factors in under-researched settings (Albania and Kosovo) [47]. A pattern of moderate risk in participant selection and outcome measurement produced a moderate overall classification. The study illustrates contextual challenges in school-based CBT implementation [29]. Moderate risk in participant selection and outcome measurement informed its moderate classification, while adding insight into stakeholder experiences with online CBT [41]. A similar domain profile, with moderate risk for selection and outcome measurement, yielded a moderate overall judgment. The findings emphasize therapist concerns in blended therapy [35]. Low risk across nearly all domains and only one moderate rating gave this study an overall low-risk classification, making it the strongest methodological contribution among the qualitative set [30]. Moderate risks in participant selection and reporting bias resulted in a moderate overall rating, but its focus on COVID-era digital care adds contemporary relevance [43]. Mostly, moderate ratings, especially in missing data and outcome measurement, led to a moderate classification, though the NASSS framework enhances interpretability [46]. Moderate bias in participant selection and outcome measurement resulted in a moderate risk judgment, with important insights into implementation within child welfare programs [44]. Moderate ratings for participant selection and outcome measurement gave a moderate classification, yet the study provides detailed accounts from therapists and managers in chronic pain/fatigue contexts [19]. Multiple moderate domain ratings (deviations, missing data, outcome measurement) explained its moderate overall risk. Despite this, it offered comprehensive data on CALM intervention implementation [31]. Moderate risk due to participant selection and missing data resulted in a moderate classification, but the large, multisite scope strengthens generalizability [32]. Moderate risks across several domains (deviations, missing data) justified a moderate rating, though the explicit reporting of stakeholder engagement strategies bolsters rigor [49]. Mostly, low ratings with moderate concerns in participant selection and outcome measurement led to a moderate classification, and its mixed-methods approach enriches the findings [33].

The qualitative studies were categorized primarily under moderate risk of bias, reflecting consistent strengths in intervention classification, adherence, and reporting, while moderate concerns were noted across domains such as participant selection, outcome measurement, missing data, and confounding. These studies generally exhibited well-conducted intervention processes with reliable reporting, but limitations in participant selection and handling of missing data were recurring issues that introduced moderate risks. A single study was classified as serious risk of bias due to significant concerns related to confounding, participant selection, and deviations from intended interventions, which heavily impacted its validity. One study achieved a low risk of bias designation, demonstrating robust performance across all assessed domains, including intervention classification, adherence, reporting, and participant selection, ensuring high reliability.

For RCTs, the ROBINS-II tool was applied, revealing three studies with some concerns and one study with low risk of bias. Studies with some concern demonstrated strengths in areas like randomization, intervention fidelity, and outcome reporting but were limited by insufficient details on randomization sequence, allocation concealment, handling of missing data, and occasional deviations from intended interventions, introducing moderate bias. One study classified as low risk exhibited rigorous methodology, effectively addressing critical domains, such as randomization, blinding, handling of missing data, and comprehensive reporting, ensuring high internal validity. RCT of CBT for chronic fatigue syndrome in mental health care was rated as having some concerns. While randomization was reported, details of allocation concealment and missing data handling were insufficient. Nonetheless, the study contributed important evidence on cost-effectiveness and outcomes of implementing CBT in real-world clinical services [50]. Observational analysis of a multicenter RCT on CBT for psychosis was also rated as having some concerns. Unclear reporting of randomization and incomplete handling of missing data reduced confidence, though strengths in intervention fidelity and outcome measurement supported its contribution to understanding therapeutic processes [34]. A hybrid Type 2 RCT of brief CBT in primary care was rated as having some concerns due to incomplete blinding and partial deviations from intended intervention delivery. Nevertheless, it demonstrated strengths in randomization, outcome reporting, and handling of missing data, supporting confidence in its findings on implementation feasibility [21]. Effectiveness RCT on CBT for social anxiety and panic disorder was judged to be at low risk of bias. It exhibited methodological rigor through robust randomization, allocation concealment, assessor blinding, and complete reporting, making it one of the strongest trials included in this review [36]. Randomized implementation trial of TF-CBT for adjudicated teens showed some concerns, particularly related to missing outcome data and limited blinding of assessors. Despite these limitations, the study provided valuable evidence on training strategies and sustainability challenges in residential treatment settings [22]. The risk of bias assessment results is presented in Supporting file 3.

The methodological quality and risk of bias assessment of the included studies are provided in Supporting File 3, conducted using the JBI critical appraisal tool, the MMAT, the ROBINS-I tool, and the ROBINS-II tool.

### 4.5. Intervention

The interventions analyzed in the systematic review include various forms of CBT, categorized based on their delivery and focus.

iCBT was the most frequently studied, with nine publications exploring its implementation. For example, IJzerman and van der Vaart [17] found that limited training opportunities and insufficient facilitating conditions hampered uptake in Dutch medical settings. Chan [45] studied computerized CBT for insomnia, where patient motivation and digital literacy posed major challenges despite the intervention's accessibility benefits. Wilhelmsen [37] reported similar concerns among Norwegian general practitioners, who valued structured guidance but expressed skepticism about effectiveness and cited time constraints. Doukani [47], working in Albania and Kosovo, highlighted infrastructural barriers and cultural stigma, though organizational readiness and collaboration across borders acted as enablers. In the UK, Duffy [41] identified low digital literacy and service integration challenges as barriers to iCBT, while Titzler [35] in Germany described therapist training gaps and patient resistance in blended models combining online and face-to-face sessions. Thapar [43] studied a publicly funded Canadian program and noted difficulties with engagement and privacy concerns, whereas Banck [46] in Sweden found therapist training and strong management support to be critical facilitators. Finally, van der Vaart [19] demonstrated the feasibility of guided iCBT for chronic conditions, emphasizing flexibility and organizational support.

Exposure-based CBT was the focus of Wolitzky-Taylor [20], who examined its use in US community mental health. Both providers and patients acknowledged the clinical relevance of exposure therapy, but providers cited competency gaps and training deficits as major barriers, while patients noted symptom-related difficulties in engaging with treatment.

Disorder-specific CBT approaches featured prominently in 14 studies. For example, Wittorf [34] studied CBT for psychosis in Germany and highlighted therapeutic alliance and empathy as central facilitators. Scheeres [18] tested CBT for chronic fatigue syndrome in the Netherlands, reporting feasibility and patient satisfaction as positive outcomes. Haug [36] evaluated CBT for social anxiety and panic disorders in Norway, demonstrating the effectiveness of tailored interventions, while Cully [21] implemented brief CBT for cardiopulmonary patients in the US, identifying time constraints and stigma as barriers. Several US studies, including Cohen [22] on TF-CBT in youth justice settings, Shepardson [28] on integrated anxiety interventions, and Stirman [33] on community-based clinicians, further demonstrated how organizational support, supervision, and clinician engagement facilitated implementation.

TF-CBT was a recurring theme in multiple contexts. Cohen [22] and Harrison [30] highlighted the importance of staff training and supportive environments in youth and community settings, while Connors [46] demonstrated how leadership buy-in and collaboration were critical to embedding TF-CBT in school-based services. Valentine [49] further extended this by showing that leadership support and tailored training improved uptake of TF-CBT in safety-net hospital clinics.

Other innovative models included stepped-care approaches, as examined by Kopelovich [29] in community care for psychosis and Savard [42] in insomnia among cancer patients, both of which reported feasibility and organizational readiness as key facilitators. Dimitropoulos [44] explored a transdiagnostic CBT model for child welfare settings, emphasizing trauma-informed care and interdisciplinary collaboration.

Together, these intervention-specific findings illustrate that while the format and target population varied, common themes of clinician training, organizational commitment, and flexibility in delivery consistently influenced implementation success.

### 4.6. Barriers to and Facilitators of Implementing and Using iCBT

**Figure 3:**
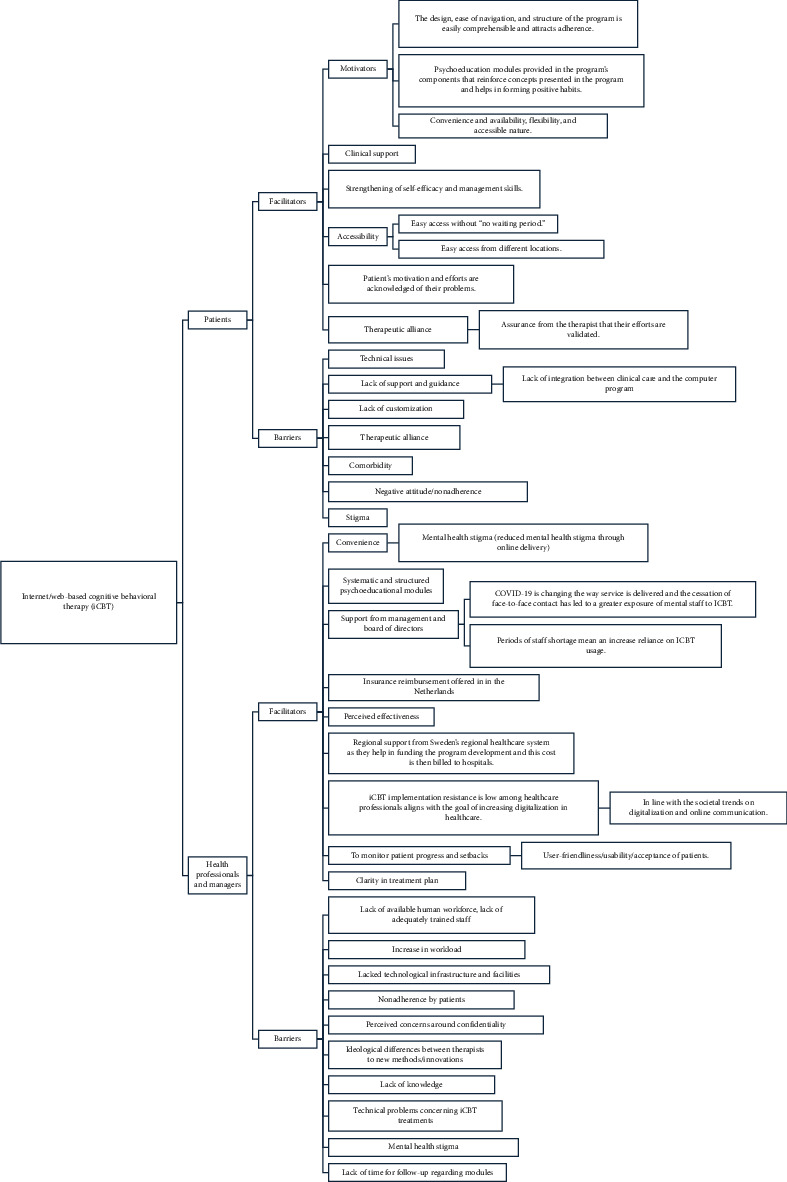
Barriers and facilitators of implementing internet-based/web-based CBT.

Nine studies specifically examined the barriers and facilitators influencing the implementation of iCBT across various settings. The results are summarized below in Tables 2, 3, and Figure 3. In the Netherlands, IJzerman and van der Vaart [17] highlighted restricted access to iCBT platforms and inadequate training opportunities as major obstacles. Similarly, Wilhelmsen [37] found that Norwegian GPs valued the structured nature of guided iCBT but faced skepticism about its effectiveness and limited time to use it in practice. From Australia, Chan [45] reported that patient motivation and digital literacy were significant challenges to engagement, despite the advantages of accessibility.

Studies in lower-resource and cross-border contexts emphasized infrastructural barriers. Doukani [47], working across Albania and Kosovo, found that poor digital infrastructure and cultural stigma hindered implementation, though collaboration and organizational readiness acted as enablers. In the UK, Duffy [41] highlighted low digital literacy among patients and difficulties in service integration, while Banck [46] in Sweden reported that insufficient therapist training and organizational barriers complicated delivery.

Other iCBT studies identified facilitators that supported adoption. Titzler [35] in Germany reported that blended models combining online and face-to-face therapy were feasible when therapists received adequate training and support. Thapar [43] evaluated a publicly funded Canadian iCBT program, showing that accessibility and flexibility promoted uptake, though issues of patient engagement and privacy remained. Finally, van der Vaart [19] found that guided iCBT for chronic conditions was well received, with management support and patient engagement emerging as key facilitators.

Together, these studies show that iCBT implementation is frequently limited by technological and training barriers, but can be enhanced when supported by organizational readiness, therapist training, and adaptable program design.

### 4.7. TF-CBT

Five studies explored the implementation of TF-CBT for youth across diverse service settings. In the United States, Cohen [22] evaluated TF-CBT in residential treatment facilities for adjudicated teens and found that structured training and consistent supervision supported delivery, while barriers included staff turnover and limited organizational resources. Harrison [23] investigated the addition of self-care strategies to TF-CBT training and reported that while self-care practices improved clinician engagement, heavy caseloads, and competing demands hindered sustained implementation. Connors [24] examined TF-CBT in 13 urban public schools and identified strong leadership support and collaboration between educators and clinicians as key facilitators, whereas insufficient staff time and capacity were significant barriers. In the United Kingdom, McGuire [32] reported that access to best-evidenced trauma-focused care for young people was challenged by resource shortages and clinician skepticism, but supervision and interprofessional collaboration facilitated uptake. Finally, Valentine [49] studied TF-CBT adoption in US safety-net hospital clinics, demonstrating that leadership buy-in and multistakeholder engagement strengthened implementation, though organizational instability and lack of protected time for training remained obstacles.

Together, these findings show that TF-CBT implementation is shaped by a combination of organizational factors (leadership, resources, stability), provider-level factors (training, supervision, self-care, clinician attitudes), and system-level issues (service demand, policy support, interprofessional collaboration).

The results are summarized below in Tables 4 and 5, with key themes organized into organizational, provider-level, patient-level, and systemic domains.

### 4.8. Barriers of and Facilitators in Implementing and Using Exposure-Based CBT

Facilitators included provider confidence in CBT, general acceptability of the approach, and patient satisfaction with therapy, with most patients viewing treatment for anxiety as essential and endorsing gradual exposure as helpful. Minimal system-level barriers, such as transportation or appointment scheduling, were reported. However, significant barriers included inadequate training for providers in core CBT techniques like in vivo, imaginal, and interoceptive exposure, with over one-third of clinics reporting few staff trained in CBT. Many providers also perceived structured protocols as impractical and reported discomfort or lack of competence in delivering exposure therapy. On the patient side, fear of confronting avoided situations and depression-related symptoms often hindered regular treatment attendance [20].

### 4.9. Barriers of and Facilitators in Implementing and Using Transdiagnostic CBT

Barriers included high staff turnover, making it challenging to sustain knowledge about the protocol, and difficulties with training all staff due to competing priorities. Additionally, the lack of permanent caregivers for some children created challenges in engaging them fully with the intervention. Facilitators included the perceived high-quality and trauma-informed adaptability of the program, its compatibility with the existing treatment philosophy, and its ability to meet the needs of children and caregivers. Positive reception among caregivers and staff, and practical strategies for addressing core emotional and behavioral issues were key factors promoting implementation success [44].

### 4.10. Barriers of and Facilitators in Implementation of Computer-Assisted CBT

Key barriers to implementing the computer-assisted intervention included gaps in training, scheduling challenges, technical issues, competing school priorities, limited session time, staff turnover, inefficient referral processes, teachers' reluctance to sacrifice classroom time, and insufficient parental support. Facilitators included effective training and consultation, program champions who streamlined scheduling, and the program's engaging, child-friendly design. A supportive school culture, effective identification of anxious students, and strong collaboration among teachers, parents, and providers further promoted implementation, with child engagement and parental involvement playing a critical role in the program's success [29]. Provider attitudes significantly influenced the adoption of the CALM intervention, with enthusiasm varying among primary care physicians. Barriers included discomfort with mental health management, perceived low prevalence of anxiety disorders, structural clinic challenges (e.g., part-time staff, limited space, communication gaps), and patient-related issues like socioeconomic stressors, cultural resistance, and competing priorities. Facilitators included provider buy-in, low workflow burden, positive patient outcomes, and on-site mental health professionals, particularly full-time ACSs, which improved communication and integration. Pre-existing collaborative care models eased implementation, but sustaining the intervention faced challenges like funding shortages and inadequate space for therapy sessions [31].

### 4.11. Cost-Effectiveness in Implementing CBT (Facilitators)

Fifty-eight percent of patients (72 out of 125) successfully completed the treatment. Total societal and healthcare costs per patient decreased significantly after implementing CBT. From a societal perspective, costs reduced from €8030 to €6869 per patient over six months. Fatigue severity reduced, with a recovery rate of 37% of 72 patients. Productivity improved, as the mean number of real hours worked increased from 9.3 to 11.4 per week and lost productivity costs decreased. Improvements in patients' health-related quality of life (HRQoL) were observed, with the mean utility score rising from 0.57 to 0.65 [18].

### 4.12. Barriers of and Facilitators in Implementing and Using Traditional CBT

Table 6 provides an organized overview of the facilitators and barriers across the various studies on CBT implementation.

Fourteen studies investigated the implementation of traditional, disorder-specific CBT across a variety of mental health conditions and service contexts. Several common barriers emerged across these studies. At the organizational level, resource shortages and limited infrastructure were frequently reported. For example, Scheeres [18] highlighted that while CBT for chronic fatigue syndrome was cost-effective, high staff workload and limited clinic capacity restricted access. Similarly, Williams [39] found that clinicians working in assertive outreach teams faced role conflicts and skepticism about CBT, which reduced integration into routine practice. Hazell [38] also reported that stigma, lack of training, and difficulties tailoring CBT to auditory hallucinations hindered dissemination.

Provider-level barriers centered on training, competence, and workload pressures. Shepardson [27] observed that primary care clinicians were often unfamiliar with CBT techniques, and competing priorities limited their ability to adopt them. Stirman [33] noted similar challenges in community mental health, where clinicians expressed concerns about fidelity and confidence in applying CBT models. Wittorf [34] further demonstrated that while therapy skills contributed to positive patient outcomes in CBT for psychosis, insufficient training and inconsistent supervision reduced clinician effectiveness.

Patient-level barriers were also prominent. Haug [36] showed that engagement in CBT for social anxiety and panic disorder was influenced by the strength of the therapeutic alliance, with dropout rates higher when rapport was weak. Savard [42], in the context of CBT for insomnia among cancer patients, reported that patient fatigue, competing treatment demands, and motivational factors limited adherence.

At the same time, multiple facilitators were identified that promoted successful CBT adoption. Organizational support and leadership played a critical role: Kopelovich [25] found that stepped-care models for psychosis were more feasible when supported by strong managerial commitment and designated coordinators. Blonigen [30] demonstrated that within the Veterans Health Administration, leadership engagement and integration into existing services enhanced uptake of CBT for criminogenic thinking. Provider-level facilitators included training opportunities, supervision, and collaboration across disciplines, as reported by Ringle [28] in youth anxiety treatment and Xanidis and Gumley [40] in CBT for psychosis. Patient-level facilitators, such as motivation and belief in treatment efficacy, also improved engagement, as described by Savard [42] and Haug [36].

Taken together, these studies indicate that the success of traditional CBT implementation depends on a balance between organizational readiness and leadership, provider competence and workload, and patient motivation and engagement.

Table 7 gives an insight into barriers and facilitators of implementing CBT, and it is coded according to the CFIR.

## 5. Discussion

This review provides a comprehensive synthesis of patients', healthcare providers', and administrators' perspectives about barriers/facilitators of implementing CBT. The most common facilitators were provider buy-in, leadership and administrative support, supportive environments, alignment with organizational goals, and stakeholder engagement. The most common barriers were lack of funding, limited time, inadequate training, workload and high staff turnover, and stigma.

One of the most pervasive barriers across diverse healthcare settings is the lack of sufficient resources. Financial constraints, inadequate funding, and limited access to mental health services significantly compromise the ability to implement evidence-based interventions like CBT. Access to professional help for mental disorders like depression is heavily influenced by individual socioeconomic status and worsened by living in low-income countries, creating a double burden [51]. In 2016, the National Mental Health Survey (NMHS) was conducted in India, the largest survey reported survey of mental morbidity. A survey estimated that nearly 150 million individuals suffer from one or the other mental morbidity in India [52]. In India, for instance, the treatment gap for common mental disorders remains as high as 80.4%, with rural and marginalized populations facing the greatest disparities. The implementation of CBT faces significant barriers and facilitators, particularly within healthcare systems where mental health resources are unevenly distributed. Limited funding and infrastructure constraints further hinder the effective rollout of evidence-based psychological interventions like CBT. However, recent policy initiatives, such as the National Mental Health Programme (NMHP) and Ayushman Bharat Digital Mission (ABDM), indicate a shift toward integrating mental health into primary care, which can serve as a facilitator for widespread CBT implementation [53, 54]. Additionally, technological interventions like Tele MANAS, including telehealth and mobile-based mental health platforms, have shown promise in expanding access to therapy, particularly in regions with inadequate mental health professionals [55]. The shift toward online modalities such as iCBT has introduced additional barriers. Although digital platforms can potentially extend the reach of CBT, challenges such as low digital literacy, technical malfunctions, and limited infrastructure (especially in lower-middle-income countries [LMIC]) impede the effective delivery of these interventions. Resistance to telemedicine and concerns regarding the effectiveness of virtual therapy are also prominent issues that need addressing.

Implementing CBT in routine practice involves navigating a complex landscape of barriers and facilitators that span individual, organizational, and systemic levels. A pattern similarly observed in studies of mental health and telehealth interventions like iCBT. Key barriers include insufficient training and support for practitioners, resource constraints, and resistance to change within established service structures, which can compromise treatment, fidelity, and sustainability. A recurrent theme in the literature is the scarcity of adequately trained practitioners. Insufficient preimplementation and ongoing training, combined with high staff turnover and heavy clinical workloads, compromise treatment fidelity. In addition, clinician resistance—often rooted in skepticism about new methods and the burden of additional administrative tasks—further exacerbates the difficulty in integrating CBT into established clinical workflows. Telehealth in India faces challenges such as low digital literacy, technical malfunctions, and inadequate infrastructure—issues that are analogous to difficulties in disseminating and maintaining high-quality CBT programs in low-resource settings [55].

Facilitators identified include comprehensive preimplementation training, formative research to adapt interventions to local contexts, and robust policy support. These factors are echoed in evolving mental health strategies in India, where integrated service models and policy initiatives like the NMHP have been pivotal in expanding access to quality care [52, 55] Consistent with the systematic review findings, research indicates that despite the well-established efficacy of CBT as an evidence-based intervention, its integration into routine clinical practice is frequently hindered by clinician resistance, resource limitations, and administrative challenges [11].

Successful implementation is often contingent upon the engagement of all relevant stakeholders—from clinicians and patients to policy makers and community leaders. When providers are convinced of the efficacy of CBT and are involved in the adaptation process, resistance is minimized and adherence to treatment protocols improves. Structured training programs that incorporate competency assessments and continuous supervision further reinforce provider buy-in, thereby enhancing treatment fidelity. Organizational factors, such as leadership support, structured training programs, and the presence of facilitators or champions within the clinical setting, have been identified as key enablers for successful implementation [56]. Furthermore, online adaptations of CBT, such as iCBT, have demonstrated potential in overcoming traditional barriers related to accessibility and workforce limitations. However, skepticism from healthcare professionals and a lack of standardized training for therapists remain significant obstacles [57]. In the case of TF-CBT, implementation efforts within public health systems have been successful when paired with sustained training, supervision, and policy-level support reinforces the importance of a multifaceted implementation strategy that includes preimplementation assessment, ongoing clinician training, and strong institutional support to facilitate the widespread adoption of CBT interventions [11, 58]. Barriers and facilitators significantly influence accessibility and effectiveness, and key barriers include the shortage of trained practitioners, financial constraints, and the limited integration of CBT into routine clinical practice. This aligns with the findings of this review, which also identified significant barriers to CBT implementation, including financial constraints and the need for specialized clinician training, despite its established effectiveness for anxiety and post-traumatic stress disorder. Additionally, studies indicate that adherence to iCBT is hindered by the lack of face-to-face clinician support, which many patients find essential for engagement and completion [59, 60]. Facilitators of implementation include structured training programs for clinicians, such as those that incorporate ongoing supervision and competency assessments. Stepped-care models, which introduce low-intensity CBT interventions before high-intensity treatments, have been shown to enhance accessibility and efficiency in mental health services. Evidence suggests that when frontline clinicians receive structured training with fidelity assessments, their ability to deliver CBT effectively improves, leading to better patient outcomes [60, 61].

### 5.1. Limitations

This systematic review has several limitations that should be acknowledged. First, only studies published in English were included, which may have led to language bias and the exclusion of potentially relevant research conducted in other languages. Second, despite rigorous efforts, full-text retrieval was not possible for a portion of the initially identified studies, which may have resulted in the omission of valuable insights and introduced selection bias. Additionally, the review did not incorporate grey literature, such as unpublished studies, conference proceedings, or policy documents, potentially limiting the comprehensiveness of the evidence base. Lastly, the majority of included studies originated from high-income countries, which may affect the generalizability of findings to low- and middle-income settings where implementation contexts can differ significantly.

## 6. Conclusion

This review established major barriers and facilitators that impact the implementation of CBT in various healthcare settings, with the CFIR acting as the guiding framework. The most frequently reported barriers were inadequate training, poor organizational support, time limitations, and clinician resistance to change. Facilitators included formal training programs, supportive leadership, stakeholder involvement, and flexibility in CBT delivery models. By applying the CFIR framework, this review provides a clear understanding of the multilevel determinants affecting CBT integration. These findings directly address the review question by outlining actionable factors that either hinder or support CBT adoption in routine practice. These findings can be used to guide targeted implementation interventions and capacity development efforts, especially when implementing CBT in new service environments. Nevertheless, since the geographic concentration of included studies in the high-income countries, more studies must be conducted in order to generalize these findings to low- and middle-income settings.

## Figures and Tables

**Table 1:** Characteristics of included studies.

Author, year and country	Study design	Sample size^1^	Population	Intervention	Outcome (barriers facilitators)
Renée V. H. IJzerman, Rosalie van der Vaart, 2019, Netherlands	Cross-sectional study survey	107 psychologists	107 psychologists working in medical settings	Internet-based cognitive behavioral therapy	Key barriers to implementation included limited access to iCBT^2^, lack of training, and insufficient facilitating conditions.

Kate Wolitzky-Taylor, 2019, United States	Cross-sectional survey	148 (106 providers/administrators and 42 patients)	106 providers/administrators and 42 patients in adult community mental health settings	Exposure-based cognitive behavioral therapy for anxiety disorders	Providers and administrators identified lack of training and competency as primary barriers to delivering exposure-based CBT. Patients reported that their own symptoms often impacted treatment receipt. Both groups found exposure therapy acceptable but noted its limited use in practice.

Andreas Wittorf, 2013, Germany	Observational cross-sectional study	79 patients	Patients undergoing cognitive behavioral therapy for psychosis	Cognitive behavioral therapy for psychosis	Facilitators: Empathy, therapeutic alliance, flexibility, patient-centered approach, ongoing training.

Korine Scheeres, 2008, Netherlands	Quasiexperimental	125 patients diagnosed with chronic fatigue syndrome	125 patients diagnosed with chronic fatigue syndrome	Cognitive behavioral therapy implemented in a mental health center	Facilitators: Efficacy, cost-effectiveness, satisfaction, feasibility, support system.

Jeffrey A. Cully, 2012, United states	Randomized controlled trial	320 primary care patients	320 primary care patients with chronic cardiopulmonary diseases, experiencing depression and anxiety	Brief cognitive behavioral therapy administered by clinicians in primary care settings	Barriers: Limited clinician training, patient engagement, time constraints, stigma. Facilitators: Integration into primary care, clinician support, brief therapy format, high patient acceptability.

Thomas Haug, 2016, Norway	Randomized controlled trial	82 adults	Adults diagnosed with social anxiety disorder or panic disorder	Cognitive behavioral therapy	Facilitators: Strong working alliance, therapist competence, patient engagement, tailored interventions.

Judith A. Cohen, 2017, United States	Randomized controlled trial	129 adjudicated teens	Adjudicated teens in residential treatment facilities	Trauma-focused cognitive behavioral therapy	Barriers: Trauma history, facility constraints, resistance to treatment, staff turnover. Facilitators: Evidence-based intervention, staff training, supportive environment, strong therapeutic rapport.

Julie P. Harrison, 2023, United States	Mixed method	86 community-based clinicians	115 clinicians and supervisors	Trauma-focused cognitive behavioral therapy training augmented with self-care practices	Barriers: Time constraints, burnout, lack of resources, insufficient supervision. Facilitators: Self-care integration, clinician training, supervisor support, organizational commitment.

E. H. Connors, 2021, United States	Mixed method	31 clinicians	31 clinicians in 13 urban public schools	Trauma-focused cognitive behavioral therapy	Barriers: Limited time, school staff turnover, resource constraints, competing priorities. Facilitators: Training and support, school leadership buy-in, collaboration, clinician commitment.

Sarah L. Kopelovich, 2022, United States	Mixed method	58 early adopters	Early adopters of cognitive behavioral therapy for psychosis stepped care in community mental healthcare settings	Cognitive behavioral therapy for psychosis stepped care	Early adopters perceived CBTp^3^-SC as acceptable, feasible, and appropriate. Key facilitators included organizational readiness, a CBTp coordinator role, and a desire to adapt the intervention. Barriers included the need for training and implementation support.

Erin Koffel, 2020, United States	Qualitative	17 providers	Providers in VA primary care settings	Cognitive behavioral therapy for insomnia	Barriers: Time constraints, stigma, logistical challenges.

Cassie M. Hazell, 2017, United Kingdom	Qualitative	21 individuals (participants were divided into three focus groups based on their locality, each including between 6 and 10 participants.) 124 mental health clinicians 145 participants	Individuals with lived experience of hearing voices and clinicians	Brief cognitive behavioral therapy for voices	Barriers: Stigma, variability in patient engagement, resource limitations.

Charles Chan, 2017, Australia	Qualitative	39 participants	Depressed patients in a psychiatric clinic	Computerized cognitive behavioral therapy for insomnia	Barriers: Digital literacy, motivational challenges, technical issues. Facilitators: Ease of access, personalization, perceived effectiveness.

Maja Wilhelmsen, 2014, Norway	Qualitative	11 general practitioners (GPs) from northern Norway	General practitioners (GPs)	Guided internet-based cognitive behavioral therapy for depression	Barriers: Limited time, lack of training, skepticism about effectiveness. Facilitators: Structured guidance, ease of integration, patient acceptability.

Robyn L. Shepardson, 2022, United States	Qualitative	18 behavioral health providers Psychologists: 9 (50%) Social workers: 6 (33%) Registered nurses: 3 (17%)	Behavioral health providers in integrated primary care settings	Evidence-based cognitive behavioral anxiety interventions	Barriers: High workload, limited training, resource constraints. Facilitators: Integrated care models, supportive leadership, tailored interventions.

C. H. J. Williams, 2008, United Kingdom	Qualitative	28 assertive outreach teams (AOTs)	Assertive outreach teams	Cognitive behavioral therapy	Barriers: Team resistance, role conflicts, insufficient training.

Josée Savard, 2022, Canada	Qualitative	59 participants across nine focus groups Clinicians: 43 participants, including physicians, nurses, radiation therapists, and psychologists. Administrators: 6 participants. Cancer patients: 10 participants.	Cancer patients	Stepped-care cognitive behavioral therapy for insomnia	Barriers: Patient fatigue, competing demands, resource limitations. Facilitators: Flexibility in delivery, provider training, patient motivation.

Vanesa A. Ringle, 2015, United States	Qualitative	Therapists: 50 participants	Therapists in community settings	Cognitive behavioral therapy for youth anxiety	Barriers: Time constraints, lack of training, organizational challenges. Facilitators: Evidence-based protocols, supervision support, youth engagement.

Nikos Xanidis, 2020, United Kingdom	Qualitative	14 mental health professionals. (Participants consisted of mental health nurses [*n* = 5], consultant psychiatrists [*n* = 2], clinical/counselling psychologists [*n* = 2], CBT therapists [*n* = 2], an occupational therapist [*n* = 1], a team leader [*n* = 1], and a senior adult mental health manager [*n* = 1].)	Mental health professionals	Cognitive behavioral therapy for psychosis	Barriers: Staff skepticism, role confusion, limited time. Facilitators: Normalization of practice, interdisciplinary collaboration, training.

Asmae Doukani, 2021, Albania and Kosovo	Qualitative	68 mental healthcare professionals (Participants represented a spectrum of health professionals working at and in association with CMHCs, with nursing profession accounting for the highest number of participants [25/69, 38%], followed by social workers [13/69, 19%], psychiatrists [13/69, 19%], psychologists [11/69, 16%], general practitioners (GPs) [4/69, 6%], occupational therapist [1/69, 2%], and speech and language therapist [1/69, 2%])	Community mental health services—mental healthcare professionals	Internet-based cognitive behavioral therapy for depression	Barriers: Digital infrastructure gaps, cultural stigma, resource constraints. Facilitators: Cross-border collaboration, organizational readiness, user training.

Margaret E. Crane, 2021, United States	Qualitative	45 school-based service providers	School-based mental health providers	Computer-assisted cognitive behavioral therapy for child anxiety	Barriers: Technological issues, staff workload, variability in school infrastructure. Facilitators: Child engagement tools, supportive school leadership, ease of access.

Daniel Duffy, 2023, United Kingdom	Qualitative	19 individuals Service providers: 6 Commercial iCBT representatives: 6 Patients who received a course of iCBT: 7	Stakeholders in the UK's improving access to psychological therapies program	Internet-delivered cognitive behavioral therapy	Barriers: Low digital literacy, service integration challenges, user skepticism. Facilitators: Stakeholder collaboration, adaptable platforms, organizational support.

I. Titzler, 2018, Germany	Qualitative	5 therapists	Psychotherapists	Blended psychotherapy for depression—which the clinical and cost-effectiveness of blended therapy for depression, consisting of ten internet- and mobile-based cognitive behavioral therapy modules and six face2face sessions, was compared to the treatment usually provided by general practitioners.	Barriers: Lack of therapist training, technological issues, time constraints, patient resistance. Facilitators: Therapist support, patient flexibility, integration into clinical practice, strong therapeutic relationship.

Blonigen, D. M., 2015, United States	Qualitative	22 specialists (These specialists were trained in cognitive behavioral treatments for criminogenic thinking, specifically MRT^4^ and T4C^5^)	Veterans' health administration staff	Cognitive behavioral treatments for criminogenic thinking	Barriers: Staff resistance, limited training, time constraints, stigma surrounding criminal behavior. Facilitators: Organizational support, staff buy-in, targeted training, integration with existing services.

Serena Thapar, 2022, Canada	Qualitative	30 individuals (Patients: 15 therapists: 15)	Patients and therapists in Ontario	Publicly funded internet-based cognitive behavioral therapy program	Barriers: Technology access issues, patient engagement, lack of in-person interaction, privacy concerns. Facilitators: Accessibility, flexibility, positive therapist and patient experiences, strong program support.

Josefin Kadesjö Banck, 2020, Sweden	Qualitative	12 individuals (Therapists: 7, managers: 5)	Therapists and managers in outpatient psychiatric health care	Internet-delivered cognitive behavioral therapy for insomnia	Barriers: Technological issues, patient adherence, lack of therapist training, organizational constraints. Facilitators: Strong management support, therapist training, program adaptability, patient interest in online treatment.

Gina Dimitropoulos, 2023, Canada	Qualitative	20 staff members	Children in a child welfare residential treatment program	Transdiagnostic cognitive behavioral therapy	Barriers: High staff turnover, complex trauma presentations, resource limitations. Facilitators: Trauma-informed care, interdisciplinary collaboration, strong leadership.

Rosalie van der Vaart, 2019, Netherlands	Qualitative	Total participants: 18 Therapists: 14 Managers: 4	Therapists and managers in mental health care	Guided internet-delivered cognitive behavioral therapy for chronic pain and fatigue	Barriers: Technological issues, patient adherence, lack of therapist expertise, time limitations. Facilitators: Management support, therapist training, patient engagement, flexibility of the intervention.

Geoffrey M. Curran, 2012, United States	Qualitative	Total participants: 61 anxiety clinical specialists (ACSs): 14 Primary care physicians [PCPs]: 18 Primary care nurses: 13 Clinic administrators and staff: 16	Primary care clinics—47 clinic staff members (18 primary care providers, 13 nurses, 8 clinic administrators, and 8 clinic staff) and 14 study-trained anxiety clinical specialists (ACSs) who coordinated the collaborative care and provided cognitive behavioral therapy.	Coordinated anxiety Learning and management program—a collaborative care approach for anxiety disorders.	Barriers included variable provider interest, part-time staffing, and patient socioeconomic stressors. Facilitators included low implementation burden, provider satisfaction, frequent ACS interactions, existing collaborative care practices, and on-site mental health staff.

Rosie McGuire, 2024, United Kingdom	Qualitative	243 mental health professionals	Care-experienced young people with post-traumatic stress disorder	Cognitive therapy for post-traumatic stress disorder (a type of trauma-focused cognitive behavioral therapy)	Barriers: Systemic challenges, stigma, limited specialist availability. Facilitators: Trauma-informed care, strong therapeutic alliance, multiagency collaboration.

Sarah E. Valentine, 2022, United States	Qualitative	Total of 22 participants Primary care physicians: 6 Integrated behavioral Health clinicians: 8 Community wellness advocates: 3 Clinical Leadership: 5	Stakeholders in safety-net hospital primary care clinics, including clinicians, administrators, and patients	Brief trauma-focused cognitive behavioral therapy for post-traumatic stress disorder	Barriers: Competing priorities, time constraints, limited resources. Facilitators: Stakeholder collaboration, tailored training, leadership support.

Shannon Wiltsey Stirman, 2013, United States	Qualitative	Total of 95 participants These participants included clinicians and supervisors who worked with adult mental health service consumers.	Clinicians in community mental health settings	Cognitive therapy	Barriers: High caseloads, limited training opportunities, variability in client engagement. Facilitators: Ongoing supervision, organizational support, alignment with clinician values.

^1^Total number of sample size = 2082.

^2^Internet-based cognitive behavioral therapy.

^3^Cognitive behavioral therapy for psychosis.

^4^Moral reconation therapy.

^5^Thinking for a change.

**Table 2:** Barriers to implementing and using iCBT—health professionals and managers' perspective and patients' perspective.

	Barrier category	Description
Barriers to iCBT implementation and usage—health professionals and managers' perspective	Access and organizational issues	Limited access for psychologists; organizational resistance to implement iCBT; low usage rates. Limited time for sessions inefficient referral processes
	Knowledge and training	Lack of training and knowledge; high competence standards required; limited practical training.
	Time constraints	Time-intensive nature of new methods like iCBT; limited time for follow-ups on modules.
	Technological issues	Immature technology; lack of interoperability with patient management systems; technical disruptions.
	Cultural and attitudinal issues	Resistance from general practitioners and therapists; conflicts with standard practices; skepticism.
	Resource constraints	No funding solutions for online services; lack of digital competence in the organization.
	Patient-related barriers	Low frequency of suitable candidates; inconsistent patient engagement; stigma; lack of follow-ups.
	System integration	Not part of regular care; unclear embedding into healthcare systems; bureaucratic limitations.
	Sustainability	Lack of funding to retain anxiety clinical specialists [ACSs] and maintain the program Limited space for therapy sessions
	Parent involvement	Lack of parental support failure to assist with therapy homework

Barriers to iCBT implementation and usage—patients' perspective	Technical issues	Poor integration with clinical care; low technical affinity; technical problems causing interruptions.
	Stigma and suitability	Mental health stigma; unsuitability for elderly or cognitively impaired patients.
	Engagement and motivation	Reservations; low adherence; difficulty in building therapeutic rapport online.
	Customization and accessibility	Limited customization; low added value for patients with prior therapy experience.

**Table 3:** Facilitators of implementing and using iCBT—health professionals and managers' perspective and patients' perspective.

	Facilitator category	Description
Facilitators to iCBT implementation—health professionals and managers' perspective	Convenience and flexibility	Online modules provide flexibility and structured approaches; time and cost-effective.
	Supportive organizational culture	Digital innovation welcomed; support from management and regional health authorities.
	Training and education	High-quality training programs; opportunities for skill enhancement; therapeutic skill development. Useful training sessions, supportive consultation sessions.
	System-level benefits	Insurance reimbursement; alignment with societal digitalization trends.
	Patient monitoring	Ability to track patient progress; clarity in treatment protocols; structured and systematic care.
	Patient accessibility	Reaching underserved populations; reduced waiting times; greater accessibility in rural areas.
	Collaboration	Supportive school culture effective identification of anxious youth strong collaboration between teachers, parents, and providers
	Motivational factors	User-friendly design; psychoeducation reinforcing positive habits; constant availability.
	Clinical support	Therapist reviews create accountability; positive feedback encourages adherence.

Facilitators to iCBT implementation and usage—patients' perspective	Convenience and accessibility	Location and time independence; no waiting periods.
	Empowerment	Strengthened self-management skills; ability to work at one's own pace.

**Table 4:** Summary of key barriers and facilitators.

Domain	Facilitators	Barriers
Training and support	W + L training improved fidelity, engagement, and screening rates.	Web-only training had limited effectiveness.
Leadership and admin	Strong leadership and interagency collaboration enhanced resource allocation and supervision.	Weak leadership failed to prioritize evidence-based practices.
Therapist competency	91% reported increased confidence; 86% improved stress management through mindfulness.	Anxiety, self-doubt, and avoidance of trauma narratives impeded effective delivery.
Patient engagement	Tailored interventions and stakeholder engagement increased acceptability.	Stigma, mistrust, and environmental instability reduced engagement and therapy continuity.
Systemic issues	Flexible workflow design and structured resources minimized logistical hurdles.	Scheduling conflicts, workload imbalances, and poor care team coordination disrupted continuity.

**Table 5:** Summary of barriers and facilitators by key metrics.

Metric	Facilitators	Barriers
Completion rates	Higher with W + L training (48% vs. 22%).	Dropout and avoidance among clients.
Fidelity maintenance	Improved with post-training support.	Inconsistent session numbers; weak supervision.
Screening and engagement	Licensed therapists excelled, especially in W + L groups.	Ineffective web-only training.
Organizational efficiency	Tailored processes streamlined workflows.	High workloads and insufficient resources.

**Table 6:** Overview of the facilitators and barriers to traditional CBT implementation.

Intervention	Facilitators	Barriers
CBT for psychosis (CBTp)	Feedback, agenda setting, therapeutic alliance, patient empowerment, competence, patient engagement	Mixed effects of guided discovery
Brief CBT in primary care	Flexible protocols, training, stakeholder engagement	Time constraints, variable clinician expertise, patient stigma, balancing fidelity with customization
CBT for panic and social anxiety disorders	Early competence, stable therapeutic alliance, regular supervision	Variability in baseline social functioning
CBT for psychosis in specialty clinics	Leadership support, clinician training, informal adaptations	Resource constraints, high caseloads, systemic issues
CBT-I in primary care	Cost-effectiveness, improved sleep outcomes, stakeholder training	Limited physician knowledge, workflow challenges, patient resistance
Evidence-based CBT for anxiety in integrated primary care (IPC)	Simple, quick interventions, user-friendly resources, motivated providers	Limited time, inadequate training, patient engagement challenges
CBT for psychosis	Leadership advocacy, training, tailoring to patient needs	Resource limitations, medical model dominance, team misalignment
Youth anxiety CBT	Workplace support, supervision, adaptable delivery methods	Psychosocial stressors, low motivation, intervention rigidity
CBT in veterans justice program (VJP)	Patient incentives, peer support, tailored interventions, advocacy partnerships	Time and resource constraints, stigma, bias, resource gaps, low motivation, uncertainties regarding sustained funding for training, materials, and ongoing support.
Cognitive therapy (CT)	Alignment with organizational goals, supportive environments, structured approach	Clinician resistance, workload, concerns about applicability
CBT-I implementation	Stakeholder training, leadership support, nonpharmacological benefits	Poor familiarity with guidelines, patient preference for medication, system inefficiencies
Evidence-based anxiety interventions	Brief effective interventions, simplified tools improving engagement and providing confidence	Short sessions, training gaps, patient motivation
CBT for psychosis (CBTp)	Strong leadership, improved training, promoting empowering benefits	Severe symptoms, limited family or peer support, limited resources, lack of staff awareness, insufficient training and supervision, difficulties with referral pathway.

**Table 7:** Coded according to the CFIR.

Author (year), Country	Barriers coded by the CFIR	Facilitators coded by the CFIR
IJzerman (2019), Netherlands	Characteristics of individuals: Limited access to iCBT; low current usage rates. Inner setting: Lack of facilitating conditions [e.g., resources, training]	Characteristics of individuals: High behavioral intention to use iCBT; Inner setting: Positive performance expectancy (belief in iCBT's effectiveness). Outer setting: Social influence (peer support and norms)

Wolitzky-Taylor (2019), United States	Characteristics of individuals: Limited provider confidence and skill in delivering exposure-based CBT. Inner setting: Time constraints, lack of organizational support. Outer setting: Stigma and patient reluctance to engage in exposure therapy	Characteristics of individuals: Provider openness to training and new practices; inner setting: Positive attitudes toward CBT's efficacy among staff. Implementation process: Engagement strategies, including targeted training initiatives.

Wittorf (2013), Germany	Not explicitly identified in the study.	Characteristics of individuals: High therapist patient alliance. Implementation process: Patients' active engagement in therapy and perceived personal relevance.

Scheeres (2008), Netherlands	Not explicitly identified in the study.	Inner setting: Evidence of cost-effectiveness of CBT for CFS. Outer setting: High patient demand and need for effective CFS treatments.

Cully (2012), United States	Inner setting: Limited time for primary care clinicians to deliver CBT; lack of dedicated mental health resources in primary care settings.	Outer setting: High prevalence of depression and anxiety in chronic disease populations, creating demand. Characteristics of individuals: Clinicians' willingness to integrate mental health care with chronic disease management.

Haug (2016), Norway	Not explicitly identified in the study.	Characteristics of individuals: Strong working alliance between therapist and patient. Intervention characteristics: Demonstrated effectiveness of CBT for social anxiety and panic disorder.

Cohen (2017), United States	Inner setting: Limited staff training and high turnover in residential facilities. Characteristics of individuals: Staff resistance to adopting trauma-focused approaches.	Characteristics of individuals: Staff willingness to improve care for adjudicated teens. Outer setting: High need for evidence-based trauma interventions in this population.

Harrison (2023), United States	Characteristics of individuals: Clinician burnout and lack of time to focus on self-care. Inner setting: Limited organizational support for self-care practices.	Characteristics of individuals: Increased clinician awareness of the importance of self-care. Intervention characteristics: Integration of self-care practices with TF-CBT training, enhancing clinician well-being and capacity.

Connors (2021), United States	Inner setting: High caseloads for clinicians; limited time for sessions. Outer setting: Challenges in engaging families and caregivers in therapy.	Inner setting: Strong administrative support within schools. Outer setting: High need for trauma-focused interventions in underserved urban school populations.

Kopelovich (2022), United States	Intervention characteristics: Complexity of the CBTp-SC model. Inner setting: Limited organizational readiness and competing priorities. Characteristics of individuals: Knowledge and beliefs: Clinicians' limited familiarity and confidence in CBTp-SC delivery. Process: Planning—inadequate resources for initial implementation.	Characteristics of individuals: Strong evidence for CBTp-SC's effectiveness. Process: Stakeholder engagement and collaborative planning. Outer setting: Support from patients and families for the therapy. Characteristics of individuals: Clinicians' motivation to learn CBTp-SC.

Koffel (2020), United States	Inner setting: Limited availability of trained providers; competing demands on time. Outer setting: Challenges in patient engagement and follow-up. Characteristics of individuals: Provider uncertainty about using CBT-I tools in primary care settings.	Not explicitly identified in the study.

Hazell (2017), United Kingdom	Intervention characteristics: Perceived oversimplification of the brief CBT model; concerns about efficacy for complex cases. Characteristics of individuals: Clinician resistance due to lack of confidence or training; skepticism from individuals with lived experience. Inner setting: Time constraints in clinical practice; limited organizational support for implementing brief CBT.	Not explicitly identified in the study.

Chan (2017), Australia	Intervention characteristics Complexity: Difficulty navigating the web-based platform and technical issues. Characteristics of individuals Logistical difficulties associated with clinical visits. Stigma is associated with psychiatry. Process Planning: Lack of structured integration between clinical care and the computer program.	Intervention characteristics Design and structure of the program—data visualization improved comprehension and adherence. Accessibility Nature—the program's convenience and constant availability. Goal-oriented Nature—Focus on personal goals and motivation. Inner setting Clinical intervention and reviews—sense of accountability and motivation through clinical reviews. Outer setting Belief—patients' willingness and trust in the program. Characteristics of individuals: Motivation: High patient determination to overcome insomnia and improve sleep quality. Psychoeducation—increased awareness of routines, habits, and causes improved commitment. Process Engaging: Proactive encouragement from clinicians to commence and persist with CCBT-I. Reinforcing concepts and forming habits through consistent reminders.

Wilhelmsen (2014), Norway	Inner setting: Limited time for GPs to integrate iCBT into practice; lack of integration with existing healthcare systems. Characteristics of individuals: Limited familiarity and confidence with iCBT among GPs.	Outer setting: Increasing prevalence of depression and need for scalable interventions. Intervention characteristics: Perceived usefulness and accessibility of iCBT for patients.

Shepardson (2022), United States	Inner setting: Time constraints within primary care appointments; competing demands on behavioral health providers (BHPs). Characteristics of individuals: Lack of confidence or familiarity with specific evidence-based techniques.	Inner setting: Integration of behavioral health services within primary care. Outer setting: High prevalence of anxiety disorders in primary care. Intervention characteristics: Perceived effectiveness of cognitive behavioral anxiety interventions.

Williams (2008), United Kingdom	Intervention characteristics: Features of CBT itself that affect implementation. Outer setting: External influences, including client needs and external policies. Inner setting: Organizational context, culture, and readiness for implementation. Characteristics of individuals: Knowledge, beliefs, and attitudes of implementers. Process: Planning, engaging, and executing implementation activities.	Not explicitly identified in the study.

Savard (2022), Canada	Intervention characteristics - Complexity: Concerns about the complexity of implementing a stepped-care model. Inner setting - Structural characteristics: Lack of resources and infrastructure to support the implementation. - Readiness for implementation: Resistance to change among staff and limited training opportunities. Characteristics of individuals - Knowledge and beliefs: Limited awareness and understanding of CBT-I among healthcare providers.	Intervention characteristics Relative advantage: Perceived benefits of a web-based format increasing accessibility for patients. Inner setting Implementation Climate: Motivation among staff to offer new services and improve patient care. Process Engaging: Inclusion of stakeholders in the implementation process and providing support throughout.

Ringle (2015), United States	Intervention characteristics - Complexity: Some therapists felt constrained by the structured nature of CBT. Inner setting - Structural characteristics: Lack of institutional support hindered implementation. - Readiness for implementation: Limited supervision and resources within organizations. Characteristics of individuals - Knowledge and beliefs: Therapists' perceptions of client complexity and external stressors as obstacles.	Intervention characteristics The structured nature of CBT was helpful for some therapists. Inner setting Supportive supervision within institutions facilitated implementation. Characteristics of individuals Knowledge and beliefs: Motivated clients facilitated the use of CBT.

Xanidis (2020), United Kingdom	Intervention characteristics - Complexity: Difficulties integrating CBTp with existing practices. Inner setting - Structural characteristics: Limited resources, high caseloads, and insufficient organizational support. - Readiness for implementation: Lack of supervision and training opportunities.	Intervention characteristics Relative advantage: Recognized potential of CBTp to improve patient outcomes. Inner setting Implementation climate: Presence of leaders and champions supporting CBTp. Process Engaging: Stakeholder involvement in implementation.

Doukani (2021), Albania and Kosovo	Intervention characteristics - Complexity: Concerns about the integration of iCBT into existing clinical workflows. Inner setting - Structural characteristics: Limited technological infrastructure and human resources. - Readiness for implementation: High caseloads and lack of training opportunities. Characteristics of individuals - Knowledge and beliefs: Mental health stigma affecting help-seeking behaviors.	Intervention characteristics Relative advantage: Perceived potential of iCBT to increase access to treatment for geographically isolated individuals and reduce stigma associated with mental health care. Process Engaging: High interest and capability in receiving training for iCBT among healthcare professionals.

Crane (2021), United States	Intervention characteristics - Complexity: Challenges in integrating CCBT into existing school schedules and curricula. Inner setting - Structural characteristics: Limited access to necessary technology and private spaces for conducting sessions. - Readiness for implementation: Insufficient training and support for school-based mental health providers. Characteristics of individuals - Knowledge and beliefs: Varied perceptions among staff regarding the effectiveness of CCBT for child anxiety.	Intervention characteristics Relative advantage: Recognition of CCBT's potential to increase accessibility and engagement for students. Inner setting Implementation climate: Support from school administration and collaboration among staff members. Process Engaging: Involvement of key stakeholders, including parents and teachers, in the implementation process.

Duffy (2023), United Kingdom	Inner setting: Resistance to change—reluctance among staff to adopt new digital tools. Characteristics of individuals: Low digital literacy: Challenges for both staff and patients. Implementation process: Limited training and insufficient implementation planning.	Intervention characteristics: Perceived efficacy; flexibility and accessibility of iCBT Outer setting: Stakeholder engagement; patient-centered approaches. Implementation process: Effective training programs; stakeholder collaboration.

Titzler (2018), Germany	Intervention characteristics Complexity: Challenges in integrating digital components with traditional psychotherapy. Inner setting Resource constraints: Limited time and financial resources for implementation. Characteristics of individuals Knowledge and beliefs: Concerns about the effectiveness of blended therapy and fear of technology replacing human interactions. Process Limited training: Lack of sufficient training and guidance for psychotherapists on using digital tools.	Intervention characteristics Relative advantage: Recognition of blended therapy's potential to enhance treatment efficiency and outcomes. Customizability: Ability to adapt digital components to individual patient needs. Outer setting Patient needs: Increased demand for accessible and flexible therapeutic options. Process Stakeholder involvement: Collaboration among psychotherapists, patients, and developers to refine and improve the blended therapy approach.

Blonigen (2015), United States	Intervention characteristics Complexity: Adapting (CBT) to justice-involved veterans' needs. Inner setting Resource constraints: Insufficient staff and funding. Implementation climate: Limited organizational readiness and support. Characteristics of individuals Knowledge and beliefs: Limited understanding of criminogenic thinking concepts among staff. Process Training and planning: Lack of structured training for staff to implement CBT effectively.	Intervention characteristics Perceived efficacy: Confidence in CBT's potential to address criminogenic thinking. Outer setting Patient needs and resources: Awareness of veterans' unique challenges and their impact on implementation efforts. Process Stakeholder engagement: Strong collaboration among staff, leadership, and external partners to refine and implement the program.

Thapar (2022), Canada	Intervention characteristics Usability: Challenges with platform navigation for less tech-savvy users. Outer setting Digital divide: Some patients have limited access to stable internet. Inner setting Workflow integration: Difficulty integrating the program into existing care processes. Characteristics of individuals Digital literacy: Low digital literacy among some patients and therapists. Process Implementation planning: Initial gaps in onboarding and training for therapists.	Intervention characteristics Accessibility: Increased availability of therapy, particularly during the pandemic. Perceived efficacy: Patients and therapists recognized the program's ability to deliver effective outcomes for mental health support. Outer setting Patient needs and resources: Program addressed pandemic-related barriers to accessing in-person care. Process Stakeholder involvement: Input from patients and therapists helped improve the program during implementation.

Kadesjö Banck (2020), Sweden	Intervention characteristics Complexity: Perceived difficulty integrating iCBT-i into existing workflows. Inner setting Resource constraints: Insufficient time and staffing. Readiness for implementation: Limited support for therapists. Characteristics of individuals Knowledge and beliefs: Variability in staff confidence about the effectiveness of iCBT-i. Process Training and planning: Gaps in training for therapists and managers.	Intervention characteristics Relative advantage: Recognition of iCBT-i as a time-efficient and scalable treatment. Customizability: The ability to tailor interventions to patient needs. Outer setting Patient demand: High interest in nonpharmacological insomnia treatments. Process Stakeholder engagement: Collaboration between therapists and managers supported implementation. Iterative adaptation: Flexibility in refining the program based on feedback from therapists and patients.

Dimitropoulos (2023), Canada	Intervention characteristics Complexity: Adapting the therapy to the diverse needs of children. Inner setting Resource constraints: Limited staff availability and training.	Intervention characteristics Adaptability: Flexibility in tailoring therapy to meet individual needs. Process Stakeholder engagement: Collaboration among therapists, caregivers, and program administrators.

van der Vaart (2019), Netherlands	Intervention characteristics Complexity: Challenges in managing guided iCBT workflows. Inner setting Limited technical support and organizational resources. Resistance to change among some therapists.	Intervention characteristics Perceived efficacy: Recognition of iCBT's potential to improve outcomes for chronic pain and fatigue Process Training and planning: Comprehensive therapist training and supportive implementation structures.

Curran (2012), United States	Inner setting Structural characteristics: Varied readiness across clinics. Resource constraints: Limited time for primary care providers to deliver interventions. Characteristics of individuals Mixed buy-in from staff, the level of agreement, support, or commitment from the staff is varied.	Outer setting Patient Demand: Strong demand for collaborative care models. Process Stakeholder involvement: Integration of study-trained anxiety clinical specialists (ACSs) improved implementation.

McGuire (2024), United Kingdom	Inner setting Resource constraints: Limited availability of trained professionals for delivering cognitive therapy for PTSD. Implementation Climate: High caseloads and staff burnout.	Intervention characteristics Perceived efficacy: Recognition of cognitive therapy's impact on PTSD. Process Stakeholder involvement: Collaborative efforts with care-experienced young people to tailor interventions to their needs.

Valentine (2022), United States	Intervention characteristics Complexity: Adapting trauma-focused CBT to fit the time constraints of primary care. Inner setting Workflow integration: Challenges in embedding the intervention into existing care routines. Outer setting Patient needs and resources: Addressing patient hesitancy due to stigma around mental health. Characteristics of individuals Knowledge and beliefs: Variability in staff confidence about delivering trauma-focused CBT.	Process Stakeholder engagement: Active involvement of clinicians, administrators, and patients in the development process. Intervention characteristics Adaptability: Flexibility of the intervention to suit primary care settings. Outer setting Patient demand: High interest in accessible, brief trauma-focused therapy. Process Iterative development: Continuous refinement of the intervention based on stakeholder feedback.

Stirman (2013), United States	Intervention characteristics Complexity: Perceived difficulty integrating cognitive therapy into high-demand community mental health settings. Inner setting Resource constraints: Insufficient time and organizational support. Characteristics of individuals Knowledge and beliefs: Mixed perceptions of cognitive therapy's relevance to diverse client populations. Process Training and planning: Gaps in ongoing training and access to expert supervision.	Intervention characteristics Relative advantage: Recognition of cognitive therapy's effectiveness in improving patient outcomes. Outer setting Patient demand: Strong demand for evidence-based practices in mental health care. Process Stakeholder involvement: Involvement of clinicians in adapting cognitive therapy to meet specific client needs. Training support: Provision of structured training programs and tools to enhance clinician confidence and skill.

## Data Availability

The original contributions presented in this study are included in the article. Further inquiries can be directed to the corresponding author.

## Supporting Information

Supporting Information 1 Supporting File 1: PROSPERO Registration: This file contains the PROSPERO registration record for the systematic review, including details of the review protocol as registered with the International Prospective Register of Systematic Reviews (PROSPERO).

Supporting Information 2 Supporting File 2: Search Strategy: This file provides a comprehensive description of the search strategy used in the systematic review. It outlines the databases searched (PubMed, CINAHL, ProQuest, Web of Science, and Scopus), the time period covered (1994–2024), and the complete search strings employed.

Supporting Information 3 Supporting File 3: Risk of Bias and Quality Assessment: This file presents the methodological quality and risk of bias assessment of the included studies, conducted using the Joanna Briggs Institute (JBI) critical appraisal tool, the Mixed Methods Appraisal Tool, the ROBINS-I tool, and the ROBINS-II tool. It includes individual ratings, justifications for each judgment, and summary assessments for all included studies.
