# Supplementary material for: Barriers and Facilitators of Implementing Cognitive Behavioral Therapy: A Systematic Review Based on the Consolidated Framework for Implementation
Source: Scientifica (Cairo). 2025 Nov 10;2025:2693791. doi: 10.1155/sci5/2693791 (PMC12623086; doi:10.1155/sci5/2693791)
Supplement: Supporting Information 1 — Supporting File 1: PROSPERO Registration: This file contains the PROSPERO registration record for the systematic review, including details of the review protocol as registered with the International Prospective Register of Systematic Reviews (PROSPERO). [file 2693791.f1.pdf]

Barriers and facilitators of implementing cognitive behavior therapy: A systematic review based on the consolidated framework for implementation research

Varshini R J , Rajesh Kamath, Edlin Glane Mathias, Sanjay P Patil, R Sai Bhavana

To enable PROSPERO to focus on COVID-19 submissions, this registration record has undergone basic automated checks for eligibility and is published exactly as submitted. PROSPERO has never provided peer review, and usual checking by the PROSPERO team does not endorse content. Therefore, automatically published records should be treated as any other PROSPERO registration. Further detail is provided here.

Citation
Varshini R J , Rajesh Kamath, Edlin Glane Mathias, Sanjay P Patil, R Sai Bhavana. Barriers and facilitators of implementing cognitive behavior therapy: A systematic review based on the consolidated framework for implementation research. PROSPERO 2024 Available from https://www.crd.york.ac.uk/prospero/display_record.php?ID=CRD42024570477

PREVIEW

REVIEW TITLE AND BASIC DETAILS

Review title
Barriers and facilitators of implementing cognitive behavior therapy: A systematic review based on the consolidated framework for implementation research

Original language title
English

Review objectives
What are the primary barriers to implementing cognitive behavior therapy (CBT)?
What are the key facilitators that support the successful implementation of cognitive behavior therapy (CBT) as identified through the CFIR framework?
How do the CFIR domains (e.g., intervention characteristics, outer setting, inner setting, characteristics of individuals, and process) influence the implementation of cognitive behavior therapy (CBT)?

Keywords
Barriers, Cognitive behavioral therapy, Facilitators, Implementing/Implementation

SEARCHING AND SCREENING

Searches
The search strategy aims to find published studies. A three-step search strategy will be utilized in this review. An initial limited search of PubMed (MEDLINE), CINAHL(EBSCO), EMBASE(Elsevier), Web of Science(Clarivate), PROQUEST(Clarivate), Ovid, and Scopus(Elsevier) will be undertaken, followed by an analysis of the text words contained in the title and abstract and of the index terms used to describe articles. Studies published in English will be considered for inclusion in this review. Studies published from Jan 1, 1994 - Jan 1, 2024 will be considered for inclusion in this review.
Keywords and search terms - ("barrier"[All Fields] OR "barrier s"[All Fields] OR "barriers"[All Fields]) AND ("facilitate"[All Fields] OR "facilitated"[All Fields] OR "facilitates"[All Fields] OR "facilitating"[All Fields] OR "facilitation"[All Fields] OR "facilitations"[All Fields] OR "facilitative"[All Fields] OR "facilitator"[All Fields] OR "facilitator s"[All Fields] OR "facilitators"[All Fields]) AND ("implement"[All Fields] OR "implemented"[All Fields] OR "implementing"[All Fields] OR "implements"[All Fields]) AND ("cognitive behaviour therapy"[All Fields] OR "cognitive behavioral therapy"[MeSH Terms] OR ("cognitive"[All Fields] AND "behavioral"[All Fields] AND "therapy"[All Fields]) OR "cognitive behavioral therapy"[All

Fields] OR ("cognitive"[All Fields] AND "behavior"[All Fields] AND "therapy"[All Fields]) OR "cognitive behavior therapy" [All Fields])

Study design
This review will include qualitative, quantitative studies, and mixed methodology studies.

ELIGIBILITY CRITERIA

Condition or domain being studied
Cognitive Behavior Therapy (CBT) is a well-established psychotherapeutic approach that focuses on identifying and modifying dysfunctional thoughts, beliefs, and behaviors. It is used to treat a wide range of mental health conditions, including depression, anxiety disorders, post-traumatic stress disorder (PTSD), and obsessive-compulsive disorder (OCD). CBT aims to improve emotional regulation and develop personal coping strategies that target solving current problems.

Population
Participants/Population:
Inclusion Criteria:
Population:
Patients: Individuals who are potential recipients of Cognitive Behavior Therapy (CBT) across various age groups, including both adults and adolescents.
Providers: Healthcare professionals involved in the delivery or implementation of CBT, such as psychologists, psychiatrists, clinical social workers, and counselors.
Studies conducted across various settings, including clinical, community, and institutional environments.
Research addressing the barriers and facilitators to the implementation of CBT.
Studies published from Jan 1, 1994 to Jan 1, 2024.
Exclusion Criteria:
Population:
Studies focusing solely on populations not relevant to the implementation of CBT (e.g., non-psychological or non-therapy-related conditions).
Studies that do not address barriers and facilitators to implementation of cognitive behavior therapy.

Intervention(s) or exposure(s)
Cognitive Behavioral Therapy (CBT) is an evidence-based psychotherapy with numerous applications across various populations and conditions. Despite its effectiveness, research suggests challenges in implementing CBT in real-world settings, potentially limiting its public health impact. This systematic review aims to explore these challenges and facilitators. We will examine studies investigating the implementation of CBT across diverse contexts and settings. This review seeks to overview the critical barriers and factors that promote the successful integration of CBT into real-world settings. This knowledge can inform strategies to improve access to and effectiveness of CBT interventions for various populations.

Comparator(s) or control(s)
Not applicable

Context
Setting:
Clinical Settings: Studies conducted in various healthcare environments where Cognitive Behavior Therapy (CBT) is delivered, including hospitals, mental health clinics, private practices, and outpatient therapy centers.
Community Settings: Research involving the implementation of CBT in community-based settings, such as schools, community health centers, and social service agencies.
Institutional Settings: Studies from institutions such as long-term care facilities where CBT might be implemented.
Public Health Settings: Research focused on integrating CBT into broader public health initiatives and programs aimed at mental health promotion and disease prevention.
Populations:
General Populations: Studies that explore barriers and facilitators related to implementing CBT for a broad range of mental health conditions across different age groups and demographic characteristics.
Specific Populations: Research focusing on particular groups, such as children and adolescents, adults, elderly individuals, or populations with specific mental health disorders like anxiety, depression, or PTSD.
Other Relevant Characteristics:

Healthcare Providers: Studies that examine barriers and facilitators from the perspective of various healthcare providers involved in delivering CBT, including psychologists, psychiatrists, social workers, counselors, and therapists.
Training and Professional Development: Research that evaluates the effectiveness of training programs, professional development initiatives, or educational interventions aimed at enhancing the implementation of CBT.
Implementation Strategies: Studies investigating specific strategies, policies, or organizational supports designed to promote the adoption and integration of CBT into practice.
Geographical Scope: There are no specific restrictions based on geographical location; however, studies that provide relevant, generalizable findings across different settings and populations are preferred.

OUTCOMES TO BE ANALYSED

Main outcomes

Barriers and facilitators in implementing cognitive behavior therapy across different populations and settings.

Additional outcomes

Not applicable

DATA COLLECTION PROCESS

Data extraction (selection and coding)

A predesigned excel sheet will be prepared by the authors. Data will be extracted from papers included in the review using the standardized data extraction tool. The data extracted will include specific details about the intervention, populations, study methods, and outcomes that are significant to the review question and specific objective.

Risk of bias (quality) assessment

Three independent reviewers(VAR, SP, RB) will assess methodological quality utilizing the standardized JBI evaluation tool and the MMAT (Mixed Methods) appraisal instrument. Mixed method publications chosen for retrieval will be evaluated for methodological validity before inclusion in the review using the MMAT appraisal tool. Before being included in the review, qualitative papers selected for retrieval will be appraised for methodological validity using the JBI appraisal instrument. The JBI tool will evaluate the quality of both quantitative and qualitative studies.

PLANNED DATA SYNTHESIS

Strategy for data synthesis

Studies published from 1994 - 2024 will be considered for inclusion in this review. Search terms - These terms aimed to represent the primary concepts of ‘implementing’, ‘Cognitive behavior therapy’, and ‘barriers’ or ‘facilitators’. Keywords were generated for each of these concepts by examining the terminology used in review papers in the implementation literature and a thesaurus to locate synonyms. In addition, the keywords were combined with standard MeSH terms from the PubMed databases.
Screening of selected articles – Screening and selection of titles, abstracts, and full text will be conducted independently by two authors(VAR, SP) with the help of RAYYAN. If any conflicts will be resolved through discussion and by contacting the third reviewer(RB).
Data synthesis will involve the aggregation or synthesis of findings to generate a set of statements that represent that aggregation through assembling the findings rated according to their quality and categorizing them based on similarity of meaning. The findings will be presented in narrative form. These categories are then subjected to a meta-synthesis if and all required.

Analysis of subgroups or subsets

Randomized Controlled Trials (RCTs): Studies with rigorous design evaluating the impact of CBT compared to control groups.
Non-randomized Trials: Studies that include observational or quasi-experimental designs.
Studies where CBT is administered at a higher frequency or duration.
Studies where CBT is provided at a lower frequency or shorter duration.
Hospital-Based: CBT interventions conducted in clinical or hospital settings.
Community-Based: CBT interventions delivered in non-clinical settings, such as institutions, schools.
Diagnosis: Participants with different primary diagnoses, such as depression vs. anxiety disorders.
Severity: Participants with varying levels of severity in their conditions, e.g., mild vs. severe symptoms.

REVIEW AFFILIATION, FUNDING AND PEER REVIEW

Review team members

- Miss Varshini R J , MHA 2nd year, Department of Healthcare and Hospital Management, Prasanna School of Public Health, Manipal Academy of Higher Education, Manipal.
- Dr Rajesh Kamath, Assistant Professor - Senior Scale Sports Coordinator - PSPH Department of Healthcare and Hospital Management Prasanna School of Public Health, Manipal Academy of Higher Education, Manipal
- Dr Edlin Glane Mathias, Department of Health Technology and Informatics, Centre for Evidence-informed Decision-making, Prasanna School of Public Health, Manipal Academy of Higher Education, Manipal
- Dr Sanjay P Patil, MHA 2nd year, Department of Healthcare and Hospital Management, Prasanna School of Public Health, Manipal Academy of Higher Education, Manipal
- Dr R Sai Bhavana, MHA 2nd year, Department of Healthcare and Hospital Management, Prasanna School of Public Health, Manipal Academy of Higher Education, Manipal

Review affiliation
Prasanna School of Public Health, Manipal Academy of Higher Education

Funding source
Not funded

TIMELINE OF THE REVIEW

Review timeline
Start date: 05 October 2024. End date: 05 April 2025

Date of first submission to PROSPERO
04 September 2024

Date of registration in PROSPERO
14 September 2024

AVAILABILITY OF FULL PROTOCOL

Availability of full protocol
No preview available

CURRENT REVIEW STAGE

Publication of review results
The intention is to publish the review once completed.The review will be publcished in English

This review is ongoing, the link to the final report of the publication will be provided once it is published.

Not applicable

Stage of the review at this submission

Review stage	Started	Completed
Pilot work	✓	✓
Formal searching/study identification	✓	✓
Screening search results against inclusion criteria	✓	✓
Data extraction or receipt of IPD	✓	✓
Risk of bias/quality assessment	✓	✓
Data synthesis	✓	✓

Review status
The review is completed.

ADDITIONAL INFORMATION

PROSPERO version history

- Version 1.2 published on 20 Sep 2024
- Version 1.1 published on 14 Sep 2024
- Version 1.0 published on 14 Sep 2024

Review conflict of interest
None known

Country
India

Medical Subject Headings
Cognitive Behavioral Therapy; Humans; Qualitative Research

Details of any existing review of the same topic by the same authors
Not applicable

Revision note
No preview available

Disclaimer
The content of this record displays the information provided by the review team. PROSPERO does not peer review registration records or endorse their content.

PROSPERO accepts and posts the information provided in good faith; responsibility for record content rests with the review team. The owner of this record has affirmed that the information provided is truthful and that they understand that deliberate provision of inaccurate information may be construed as scientific misconduct.

PROSPERO does not accept any liability for the content provided in this record or for its use. Readers use the information provided in this record at their own risk.

Any enquiries about the record should be referred to the named review contact
