# Supplementary material for: Barriers and Facilitators of Implementing Cognitive Behavioral Therapy: A Systematic Review Based on the Consolidated Framework for Implementation
Source: Scientifica (Cairo). 2025 Nov 10;2025:2693791. doi: 10.1155/sci5/2693791 (PMC12623086; doi:10.1155/sci5/2693791)
Supplement: Supporting Information 2 — Supporting File 2: Search Strategy: This file provides a comprehensive description of the search strategy used in the systematic review. It outlines the databases searched (PubMed, CINAHL, ProQuest, Web of Science, and Scopus), the time period covered (1994–2024), and the complete search strings employed. [file 2693791.f2.docx]

**Search Strategy:**

**PubMed -** 2800

((("barrier"[All Fields] OR "barriers"[All Fields]) OR ("Facilitate"[All Fields] OR "facilitated"[All Fields] OR "facilitates"[All Fields] OR "facilitating"[All Fields] OR "facilitation"[All Fields] OR "facilitations"[All Fields] OR "facilitative"[All Fields] OR "facilitator"[All Fields] OR "facilitators"[All Fields] OR "facilitatory"[All Fields])) AND ("Implement" [All Fields] OR "implementable"[All Fields] OR "implementation"[All Fields] OR "implementations"[All Fields] OR "implemented"[All Fields] OR "implementing"[All Fields] OR "implements"[All Fields] OR "Implementation Sciences"[Text Word])) AND ("Cognition"[All Fields] OR "cognitions"[All Fields] OR "cognitive"[All Fields] OR "cognitively"[All Fields] AND "behavior"[All Fields] OR "behavioral"[All Fields] OR "behaviorally"[All Fields] OR "behaviorism"[All Fields] OR "behaviorisms"[All Fields] OR "behaviors"[All Fields] OR "behaviour"[All Fields] OR "behavioural"[All Fields] OR "behaviours"[All Fields] AND "therapeutic"[All Fields] OR "therapeutically"[All Fields] OR "therapeutics"[All Fields] OR "therapies"[All Fields] OR "therapy"[All Fields] OR "cognitive behavioral therapy"[MeSH Terms] OR "Cognition Therapy"[Text Word] OR "Cognitive Behavioral Therapies"[Text Word] OR "Cognition Therapies"[Text Word] OR "Cognitive Behavior Therapies"[Text Word] OR "Cognitive Behavior Therapy"[Text Word] OR "Cognitive Psychotherapy"[Text Word] OR "Cognitive Psychotherapies"[Text Word] OR "Cognitive Therapies"[Text Word] OR "Cognitive Behaviour Therapy"[Text Word] "Cognitive Behaviour Therapies"[Text Word] OR "Cognitive Therapy"[Text Word]

((("barrier"[All Fields] OR "barriers"[All Fields]) OR ("Facilitate"[All Fields] OR "facilitated"[All Fields] OR "facilitates"[All Fields] OR "facilitating"[All Fields] OR "facilitation"[All Fields] OR "facilitations"[All Fields] OR "facilitative"[All Fields] OR "facilitator"[All Fields] OR "facilitators"[All Fields] OR "facilitatory"[All Fields])) AND

("Implement" [All Fields] OR "implementable"[All Fields] OR "implementation"[All Fields] OR "implementations"[All Fields] OR "implemented"[All Fields] OR "implementing"[All Fields] OR "implements"[All Fields] OR "Implementation Sciences"[Text Word])) AND

"Cognition"[All Fields] OR "cognitions"[All Fields] OR "cognitive"[All Fields] OR "cognitively"[All Fields] AND "behavior"[All Fields] OR "behavioral"[All Fields] OR "behaviorally"[All Fields] OR "behaviorism"[All Fields] OR "behaviorisms"[All Fields] OR "behaviors"[All Fields] OR "behaviour"[All Fields] OR "behavioural"[All Fields] OR "behaviours"[All Fields] AND "therapeutic"[All Fields] OR "therapeutically"[All Fields] OR "therapeutics"[All Fields] OR "therapies"[All Fields] OR "therapy"[All Fields] OR "cognitive behavioral therapy"[MeSH Terms] OR "Cognition Therapy"[Text Word] OR "Cognitive Behavioral Therapies"[Text Word] OR "Cognition Therapies"[Text Word] OR "Cognitive Behavior Therapies"[Text Word] OR "Cognitive Behavior Therapy"[Text Word] OR

"Cognitive Psychotherapy"[Text Word] OR "Cognitive Psychotherapies"[Text Word] OR "Cognitive Therapies"[Text Word] OR "Cognitive Behaviour Therapy"[Text Word] "Cognitive Behaviour Therapies"[Text Word] OR "Cognitive Therapy"[Text Word] Filters: from 1994 – 2024

| S.No | Query | No. of hits | Filters | Date of search/ Time |
| --- | --- | --- | --- | --- |
| 1 | "barrier"[All Fields] | 227,396 |  | 08:14:04 |
|  | OR "barriers"[All |  |  | (IST 17:45) |
|  | Fields] |  |  |  |
|  |  |  |  | 20-9-2024 |
| 2 | "Facilitate"[All Fields] | 363,293 |  | 08:15:49 |
|  | OR "facilitated"[All |  |  | (IST 17:46) |
|  | Fields] OR |  |  | 20-9-2024 |
|  | "facilitates"[All |  |  |  |
|  | Fields] OR |  |  |  |
|  | "facilitating"[All |  |  |  |
|  | Fields] OR |  |  |  |
|  | "facilitation"[All |  |  |  |
|  | Fields] OR |  |  |  |
|  | "facilitations"[All |  |  |  |
|  | Fields] OR |  |  |  |
|  | "facilitative"[All |  |  |  |
|  | Fields] OR |  |  |  |
|  | "facilitator"[All |  |  |  |
|  | Fields] OR |  |  |  |
|  | "facilitators"[All |  |  |  |
|  | Fields] OR |  |  |  |
|  | "facilitatory"[All |  |  |  |
|  | Fields] |  |  |  |
| 3 | "Implement" [All | 434,491 |  | 08:17:09 |
|  | Fields] OR |  |  | (IST 17:47) |
|  | "implementable"[All |  |  | 20-9-2024 |
|  | Fields] OR |  |  |  |
|  | "implementation"[All |  |  |  |
|  | Fields] OR |  |  |  |
|  | "implementations"[All |  |  |  |
|  | Fields] OR |  |  |  |
|  | "implemented"[All |  |  |  |

|  | Fields] OR "implementing"[All Fields] OR "implements"[All Fields] OR "Implementation Sciences"[Text Word] |  |  |  |
| --- | --- | --- | --- | --- |
| 4 | "Cognition"[All | 6,466,391 |  | 07:33:33 |
|  | Fields] OR |  |  | (IST 17:04) |
|  | "cognitions"[All |  |  | 20-9-2024 |
|  | Fields] OR |  |  |  |
|  | "cognitive"[All Fields] |  |  |  |
|  | OR "cognitively"[All |  |  |  |
|  | Fields] AND |  |  |  |
|  | "behavior"[All Fields] |  |  |  |
|  | OR "behavioral"[All |  |  |  |
|  | Fields] OR |  |  |  |
|  | "behaviorally"[All |  |  |  |
|  | Fields] OR |  |  |  |
|  | "behaviorism"[All |  |  |  |
|  | Fields] OR |  |  |  |
|  | "behaviorisms"[All |  |  |  |
|  | Fields] OR |  |  |  |
|  | "behaviors"[All |  |  |  |
|  | Fields] OR |  |  |  |
|  | "behaviour"[All |  |  |  |
|  | Fields] OR |  |  |  |
|  | "behavioural"[All |  |  |  |
|  | Fields] OR |  |  |  |
|  | "behaviours"[All |  |  |  |
|  | Fields] AND |  |  |  |
|  | "therapeutic"[All |  |  |  |
|  | Fields] OR |  |  |  |
|  | "therapeutically"[All |  |  |  |
|  | Fields] OR |  |  |  |
|  | "therapeutics"[All |  |  |  |
|  | Fields] OR |  |  |  |
|  | "therapies"[All Fields] |  |  |  |
|  | OR "therapy"[All |  |  |  |
|  | Fields] OR "cognitive |  |  |  |
|  | behavioral |  |  |  |
|  | therapy"[MeSH |  |  |  |

|  | Terms] OR "Cognition Therapy"[Text Word] OR "Cognitive Behavioral Therapies"[Text Word] OR "Cognition Therapies"[Text Word] OR "Cognitive Behavior Therapies"[Text Word] OR "Cognitive Behavior Therapy"[Text Word] OR "Cognitive Psychotherapy"[Text Word] OR "Cognitive Psychotherapies"[Text Word] OR "Cognitive Therapies"[Text Word] OR "Cognitive Behaviour Therapy"[Text Word] "Cognitive Behaviour Therapies"[Text Word] OR "Cognitive Therapy"[Text Word] |  |  |  |
| --- | --- | --- | --- | --- |
| 5 | #1 AND #2 AND #3  AND #4 | 2800 | 1994-2024  Full text Humans English | 10-10-2024 |

**WEB OF SCIENCE** - 1,134

(((TI=(barriers)) OR TI=(barrier)) OR AB=(barriers)) OR AB=(barrier) AND

(((((((((((((((((((TI=(Facilitate)) OR TI=(facilitated)) OR TI=(facilitates)) OR TI=(facilitating)) OR TI=(facilitation)) OR TI=(facilitations)) OR TI=(facilitative)) OR TI=(facilitator)) OR TI=(facilitators)) OR TI=(facilitatory)) OR AB=(Facilitate)) OR AB=(facilitated)) OR AB=(facilitates)) OR AB=(facilitating)) OR AB=(facilitation)) OR AB=(facilitations )) OR AB=(facilitative)) OR AB=(facilitator)) OR AB=(facilitators )) OR AB=(facilitatory)

AND

(((((((((((((((TI=(Implement)) OR TI=(implementable )) OR TI=(implementation)) OR TI=(implementations )) OR TI=(implemented )) OR TI=(implementing )) OR TI=(implements

)) OR TI=(Implementation Sciences)) OR AB=(Implement )) OR AB=(implementable )) OR AB=(implementation )) OR AB=(implementations)) OR AB=(implemented )) OR AB=(implementing )) OR AB=(implements)) OR AB=(Implementation Sciences)

AND

(((((((((((((((((((((((((((((((((((((((((((((((((((((((((((TI=(Cognition)) OR TI=(cognitions )) OR

TI=(cognitive )) OR TI=(cognitively )) AND TI=(behavior)) OR TI=(behavioral )) OR TI=(behaviorally)) OR TI=(behaviorism)) OR TI=(behaviorists)) OR TI=(behaviors )) OR TI=(behaviour )) OR TI=(behavioural )) OR TI=(behaviours)) AND TI=(therapeutic )) OR TI=(therapeutically)) OR TI=(therapeutics)) OR TI=(therapies)) OR TI=(therapy)) OR TI=(cognitive behavioral therapy)) OR TI=(Cognition Therapy)) OR TI=(Cognitive Behavioral Therapies)) OR TI=(Cognition Therapies)) OR TI=(Cognitive Behavior Therapies)) OR TI=(Cognitive Behavior Therapy)) OR TI=(Cognitive Psychotherapy)) OR TI=(Cognitive Psychotherapies)) OR TI=(Cognitive Therapies)) OR TI=(Cognitive Behaviour Therapy)) OR TI=(Cognitive Behaviour Therapies)) OR TI=(Cognitive Therapy)) OR AB=(Cognition )) OR AB=(cognitions )) OR AB=(cognitive)) OR AB=(cognitively )) AND AB=(behavior )) OR AB=(behavioral )) OR AB=(behaviorally )) OR AB=(behaviorism )) OR AB=(behaviorists )) OR AB=(behaviors )) OR AB=(behaviour )) OR AB=(behavioural )) OR AB=(behaviours )) AND AB=(therapeutic)) OR AB=(therapeutically )) OR AB=(therapeutics

)) OR AB=(therapies )) OR AB=(therapy )) OR AB=(cognitive behavioral therapy)) OR AB=(Cognition Therapy)) OR AB=(Cognitive Behavioral Therapies)) OR AB=(Cognition Therapies)) OR AB=(Cognitive Behavior Therapies)) OR AB=(Cognitive Behavior Therapy)) OR AB=(Cognitive Psychotherapy)) OR AB=(Cognitive Psychotherapies)) OR AB=(Cognitive Therapies)) OR AB=(Cognitive Behaviour Therapy)) OR AB=(Cognitive Behaviour Therapies)) OR AB=(Cognitive Therapy)

| S.N  o | Query | No. of hits | Filters | Date of searc h/ Time |
| --- | --- | --- | --- | --- |
| 1 | (((TI=(barriers)) OR TI=(barrier)) OR AB=(barriers)) | 866,184 |  | 23-9- |
|  | OR AB=(barrier) |  |  | 2024 |
| 2 | (((((((((((((((((((TI=(Facilitate)) OR TI=(facilitated)) OR | 1,416,5 |  | 23-9- |
|  | TI=(facilitates)) OR TI=(facilitating)) OR | 71 |  | 2024 |
|  | TI=(facilitation)) OR TI=(facilitations)) OR |  |  |  |
|  | TI=(facilitative)) OR TI=(facilitator)) OR |  |  |  |
|  | TI=(facilitators)) OR TI=(facilitatory)) OR |  |  |  |
|  | AB=(Facilitate)) OR AB=(facilitated)) OR |  |  |  |
|  | AB=(facilitates)) OR AB=(facilitating)) OR |  |  |  |
|  | AB=(facilitation)) OR AB=(facilitations )) OR |  |  |  |

|  | AB=(facilitative)) OR AB=(facilitator)) OR AB=(facilitators )) OR AB=(facilitatory) |  |  |  |
| --- | --- | --- | --- | --- |
| 3 | (((((((((((((((TI=(Implement)) OR TI=(implementable )) OR TI=(implementation)) OR TI=(implementations )) OR TI=(implemented )) OR TI=(implementing )) OR TI=(implements )) OR TI=(Implementation Sciences)) OR AB=(Implement )) OR AB=(implementable )) OR AB=(implementation )) OR AB=(implementations)) OR AB=(implemented )) OR AB=(implementing )) OR AB=(implements)) OR AB=(Implementation Sciences) | 2,789,8  81 |  | 23-9-  2024 |
| 4 | (((((((((((((((((((((((((((((((((((((((((((((((((((((((((((TI=(Co | 2,235,3 |  | 23-9- |
|  | gnition)) OR TI=(cognitions )) OR TI=(cognitive )) OR | 90 |  | 2024 |
|  | TI=(cognitively )) AND TI=(behavior)) OR |  |  |  |
|  | TI=(behavioral )) OR TI=(behaviorally)) OR |  |  |  |
|  | TI=(behaviorism)) OR TI=(behaviorists)) OR |  |  |  |
|  | TI=(behaviors )) OR TI=(behaviour )) OR |  |  |  |
|  | TI=(behavioural )) OR TI=(behaviours)) AND |  |  |  |
|  | TI=(therapeutic )) OR TI=(therapeutically)) OR |  |  |  |
|  | TI=(therapeutics)) OR TI=(therapies)) OR |  |  |  |
|  | TI=(therapy)) OR TI=(cognitive behavioral therapy)) |  |  |  |
|  | OR TI=(Cognition Therapy)) OR TI=(Cognitive |  |  |  |
|  | Behavioral Therapies)) OR TI=(Cognition Therapies)) |  |  |  |
|  | OR TI=(Cognitive Behavior Therapies)) OR |  |  |  |
|  | TI=(Cognitive Behavior Therapy)) OR TI=(Cognitive |  |  |  |
|  | Psychotherapy)) OR TI=(Cognitive Psychotherapies)) |  |  |  |
|  | OR TI=(Cognitive Therapies)) OR TI=(Cognitive |  |  |  |
|  | Behaviour Therapy)) OR TI=(Cognitive Behaviour |  |  |  |
|  | Therapies)) OR TI=(Cognitive Therapy)) OR |  |  |  |
|  | AB=(Cognition )) OR AB=(cognitions )) OR |  |  |  |
|  | AB=(cognitive)) OR AB=(cognitively )) AND |  |  |  |
|  | AB=(behavior )) OR AB=(behavioral )) OR |  |  |  |
|  | AB=(behaviorally )) OR AB=(behaviorism )) OR |  |  |  |
|  | AB=(behaviorists )) OR AB=(behaviors )) OR |  |  |  |
|  | AB=(behaviour )) OR AB=(behavioural )) OR |  |  |  |
|  | AB=(behaviours )) AND AB=(therapeutic)) OR |  |  |  |
|  | AB=(therapeutically )) OR AB=(therapeutics )) OR |  |  |  |
|  | AB=(therapies )) OR AB=(therapy )) OR |  |  |  |
|  | AB=(cognitive behavioral therapy)) OR AB=(Cognition |  |  |  |
|  | Therapy)) OR AB=(Cognitive Behavioral Therapies)) |  |  |  |
|  | OR AB=(Cognition Therapies)) OR AB=(Cognitive |  |  |  |
|  | Behavior Therapies)) OR AB=(Cognitive Behavior |  |  |  |

|  | Therapy)) OR AB=(Cognitive Psychotherapy)) OR AB=(Cognitive Psychotherapies)) OR AB=(Cognitive Therapies)) OR AB=(Cognitive Behaviour Therapy)) OR AB=(Cognitive Behaviour Therapies)) OR AB=(Cognitive Therapy) |  |  |  |
| --- | --- | --- | --- | --- |
| 5 | #1 OR #2 AND #3 AND #4 | 1,134 | 1994- | 23-9- |
|  |  |  | 2024 | 2024 |
|  |  |  | Articl | 11:52 |
|  |  |  | e |  |
|  |  |  | Englis |  |
|  |  |  | h |  |

**PROQUEST -** 2192

((NOFT(barrier) OR NOFT(barriers) OR NOFT(Facilitate) OR NOFT(facilitated) OR NOFT(facilitates) OR NOFT(facilitating) OR NOFT(facilitation) OR NOFT(facilitations) OR NOFT(facilitative) OR NOFT(facilitator) OR NOFT(facilitators) OR NOFT(facilitatory)) AND (NOFT(Implement) OR NOFT(implementable) OR NOFT(implementation) OR NOFT(implementations) OR NOFT(implemented) OR NOFT(implementing) OR NOFT(implements) OR TI,AB,IF("Implementation Sciences")) AND (NOFT(Cognition) OR NOFT(cognitions) OR NOFT(cognitive) OR NOFT(cognitively) AND NOFT(behavior) OR NOFT(behavioral) OR NOFT(behaviorally) OR NOFT(behaviorism) OR NOFT(behaviorisms) OR NOFT(behaviors) OR NOFT(behaviour) OR NOFT(behavioural) OR NOFT(behaviours) AND NOFT(therapeutic) OR NOFT(therapeutically) OR NOFT(therapeutics) OR NOFT(therapies) OR NOFT(therapy) OR EXACT ("cognitive behavioral therapy") OR TI,AB,IF("Cognition Therapy") OR TI,AB,IF("Cognitive Behavioral Therapies") OR TI,AB,IF("Cognition Therapies") OR TI,AB,IF("Cognitive Behavior Therapies") OR TI,AB,IF("Cognitive Behavior Therapy") OR TI,AB,IF("Cognitive Psychotherapy") OR TI,AB,IF("Cognitive Psychotherapies") OR TI,AB,IF("Cognitive Therapies") OR TI,AB,IF("Cognitive Behaviour Therapy") OR TI,AB,IF ("Cognitive Behaviour Therapies") OR TI,AB,IF("Cognitive Therapy"))) AND (at.exact("Article") AND stype.exact("Scholarly Journals") AND la.exact("ENG") AND subt.exact(("patients" OR "behavior" OR "qualitative research" OR "public health" OR "mental health" OR "health care" OR "mental disorders" OR "research" OR "focus groups" OR "mental health care" OR "mixed methods research" OR "cognitive ability" OR "counseling" OR "schools" OR "stakeholders" OR "evidence-based medicine" OR "health facilities" OR "researchers") AND "intervention") AND pd(19940101-20240101))

| S.No | Database | Query | No. of hits | Filters | Date of search/ Time |
| --- | --- | --- | --- | --- | --- |
| 1 | Proquest | NOFT(barrier) OR | 321,674 |  | 2-10- |
|  |  | NOFT(barriers)OR |  |  | 2024 |
|  |  | NOFT(Facilitate) OR |  |  |  |
|  |  | NOFT(facilitated) OR |  |  |  |
|  |  | NOFT(facilitates) OR |  |  |  |
|  |  | NOFT(facilitating) OR |  |  |  |
|  |  | NOFT(facilitation) OR |  |  |  |
|  |  | NOFT(facilitations) OR |  |  |  |
|  |  | NOFT(facilitative) OR |  |  |  |
|  |  | NOFT(facilitator) OR |  |  |  |
|  |  | NOFT(facilitators) OR |  |  |  |
|  |  | NOFT(facilitatory) |  |  |  |
| 2 |  | (NOFT(Implement) OR | 312,199 |  | 2-10- |
|  |  | NOFT(implementable) |  |  | 2024 |
|  |  | OR |  |  |  |
|  |  | NOFT(implementation) |  |  |  |
|  |  | OR |  |  |  |
|  |  | NOFT(implementations) |  |  |  |
|  |  | OR NOFT(implemented) |  |  |  |
|  |  | OR NOFT(implementing) |  |  |  |
|  |  | OR NOFT(implements) |  |  |  |
|  |  | OR |  |  |  |
|  |  | TI,AB,IF("Implementation |  |  |  |
|  |  | Sciences")) |  |  |  |
| 3 |  | NOFT(Cognition) OR | 2,154,384 |  | 2-10- |
|  |  | NOFT(cognitions) OR |  |  | 2024 |
|  |  | NOFT(cognitive) OR |  |  |  |
|  |  | NOFT(cognitively) AND |  |  |  |
|  |  | NOFT(behavior) OR |  |  |  |
|  |  | NOFT(behavioral) OR |  |  |  |
|  |  | NOFT(behaviorally) OR |  |  |  |
|  |  | NOFT(behaviorism) OR |  |  |  |
|  |  | NOFT(behaviorisms) OR |  |  |  |
|  |  | NOFT(behaviors) OR |  |  |  |
|  |  | NOFT(behaviour) OR |  |  |  |
|  |  | NOFT(behavioural) OR |  |  |  |

|  |  | NOFT(behaviours) AND NOFT(therapeutic) OR NOFT(therapeutically) OR NOFT(therapeutics) OR NOFT(therapies) OR NOFT(therapy) OR EXACT ("cognitive behavioral therapy") OR TI,AB,IF("Cognition Therapy") OR TI,AB,IF("Cognitive Behavioral Therapies") OR TI,AB,IF("Cognition Therapies") OR TI,AB,IF("Cognitive Behavior Therapies") OR TI,AB,IF("Cognitive Behavior Therapy") OR TI,AB,IF("Cognitive Psychotherapy") OR TI,AB,IF("Cognitive Psychotherapies") OR TI,AB,IF("Cognitive Therapies") OR TI,AB,IF("Cognitive Behaviour Therapy") OR TI,AB,IF ("Cognitive Behaviour Therapies") OR TI,AB,IF("Cognitive Therapy") |  |  |  |
| --- | --- | --- | --- | --- | --- |
|  |  | #1 OR #2 AND #3 | 2,192 | 1994-01-01 | 23-9- |
|  |  |  | [Original | - 2024-01- | 2024 |
| 4 |  |  | database] | 01 |  |
|  |  |  |  | Scholarly |  |
|  |  |  |  | Journals |  |
|  |  |  |  | Article |  |
|  |  |  |  | intervention |  |
|  |  |  |  | AND |  |
|  |  |  |  | (patients |  |

|  |  |  |  | OR  behavior OR  qualitative research OR public health OR mental health OR health care OR mental disorders OR research OR focus groups OR mental health care OR mixed methods research OR cognitive ability OR counseling OR schools OR  stakeholders OR  evidence- based medicine OR health facilities OR  researchers)  English |  |
| --- | --- | --- | --- | --- | --- |

**CINAHL -** 4,253

| S.No | Database | Query | No. of hits | Filters | Date of search/ Time |
| --- | --- | --- | --- | --- | --- |
| 1 | CINAHL | barrier OR | 260,351 | 1994-2024 | 10-10- |
|  |  | barriersOR |  |  | 2024 |
|  |  | (Facilitate OR |  |  |  |
|  |  | facilitated OR |  |  |  |
|  |  | facilitates OR |  |  |  |
|  |  | facilitating OR |  |  |  |
|  |  | facilitation OR |  |  |  |
|  |  | facilitations OR |  |  |  |
|  |  | facilitative OR |  |  |  |
|  |  | facilitator OR |  |  |  |
|  |  | facilitators OR |  |  |  |
|  |  | facilitatory |  |  |  |
| 2 |  | Implement OR | 279,202 | 1994-2024 | 10-10- |
|  |  | implementable OR |  |  | 2024 |
|  |  | implementation OR |  |  |  |
|  |  | implementations OR |  |  |  |
|  |  | implemented OR |  |  |  |
|  |  | implementing OR |  |  |  |
|  |  | implements OR |  |  |  |
|  |  | "Implementation |  |  |  |
|  |  | Sciences" |  |  |  |
| 3 |  | Cognition OR | 2,520,142 | 1994-2024 | 10-10- |
|  |  | cognitions OR |  |  | 2024 |
|  |  | cognitive OR |  |  |  |
|  |  | cognitively AND |  |  |  |
|  |  | behavior OR |  |  |  |
|  |  | behavioral OR |  |  |  |
|  |  | behaviorally OR |  |  |  |
|  |  | behaviorism OR |  |  |  |
|  |  | behaviorisms OR |  |  |  |
|  |  | behaviors OR |  |  |  |
|  |  | behaviour OR |  |  |  |
|  |  | behavioural OR |  |  |  |
|  |  | behaviours AND |  |  |  |

|  |  | therapeutic OR therapeutically OR therapeutics OR therapies OR therapy OR (MH "cognitive behavioral therapy+") OR "Cognition Therapy" OR "Cognitive Behavioral Therapies" OR "Cognition Therapies" OR "Cognitive Behavior Therapies" OR "Cognitive Behavior Therapy" OR "Cognitive Psychotherapy" OR "Cognitive Psychotherapies" OR "Cognitive Therapies" OR "Cognitive Behaviour Therapy "Cognitive Behaviour Therapies"" OR "Cognitive Therapy")) |  |  |  |
| --- | --- | --- | --- | --- | --- |
| 4 |  | #1 OR #2 AND #3 | 4,253 | 1994-2024 |  |
|  |  | AND |  | Limited to |  |
|  |  |  |  | Article |  |
|  |  |  |  | ***Limited*** |  |
|  |  |  |  | ***to*** Human |  |
|  |  |  |  | 24,986 |  |
|  |  |  |  | ***Limited*** |  |
|  |  |  |  | ***to*** Humans |  |

|  |  |  |  | 19,602  ***Limited to*** Article  18,746  ***Limited to*** English |  |
| --- | --- | --- | --- | --- | --- |
